# Identification of the new prenyltransferase Ubi-297 from marine bacteria and elucidation of its substrate specificity

**DOI:** 10.3762/bjoc.18.72

**Published:** 2022-06-22

**Authors:** Jamshid Amiri Moghaddam, Huijuan Guo, Karsten Willing, Thomas Wichard, Christine Beemelmanns

**Affiliations:** 1 Chemical Biology Leibniz Institute for Natural Product Research and Infection Biology e.V., Hans-Knöll-Institute, Beutenbergstraße 11a, 07745 Jena, Germany; 2 Bio Pilot Plant, Leibniz Institute for Natural Product Research and Infection Biology e.V., Hans-Knöll-Institute, Beutenbergstraße 11a, 07745 Jena, Germany; 3 Institute for Inorganic and Analytical Chemistry, Friedrich Schiller University Jena, Lessingstr 8, 07743 Jena, Germany; 4 Biochemistry of Microbial Metabolism, Institute of Biochemistry, Leipzig University, Johannisallee 21–23, 04103 Leipzig, Germany

**Keywords:** Flavobacteria, prenylation, Saccharomonospora, UbiA-like prenyltransferase

## Abstract

Aromatic prenylated metabolites have important biological roles and activities in all living organisms. Compared to their importance in all domains of life, we know relatively little about their substrate scopes and metabolic functions. Here, we describe a new UbiA-like prenyltransferase (Ptase) Ubi-297 encoded in a conserved operon of several bacterial taxa, including marine Flavobacteria and the genus *Sacchromonospora*. In silico analysis of Ubi-297 homologs indicated that members of this Ptase group are composed of several transmembrane α-helices and carry a conserved and distinct aspartic-rich Mg^2+^-binding domain. We heterologously produced UbiA-like Ptases from the bacterial genera *Maribacter*, *Zobellia*, and *Algoriphagus* in *Escherichia coli*. Investigation of their substrate scope uncovered the preferential farnesylation of quinoline derivatives, such as 8-hydroxyquinoline-2-carboxylic acid (8-HQA) and quinaldic acid. The results of this study provide new insights into the abundance and diversity of Ptases in marine Flavobacteria and beyond.

## Introduction

Marine bacteria harbor an enormous potential to produce structurally diverse natural products, including prenylated aromatic metabolites [1–2]. Prenylation of metabolites most often confers increased biological activities due to enhanced lipophilicity, solubility, and improved binding abilities to target proteins [3]. The prenylation reaction, most often a C–C-bond-forming step between an aromatic acceptor moiety and a prenyl chain, is catalyzed by dedicated dominantly membrane-bound prenyltransferases (Ptases) [4–7]. Ptases belonging to the UbiA-superfamily are responsible for the modification of many important signaling molecules that are involved in a wide variety of crucial biological processes, such as cellular respiration, detoxification, and photosynthesis, within almost all living organisms [8].

**Figure 1:**
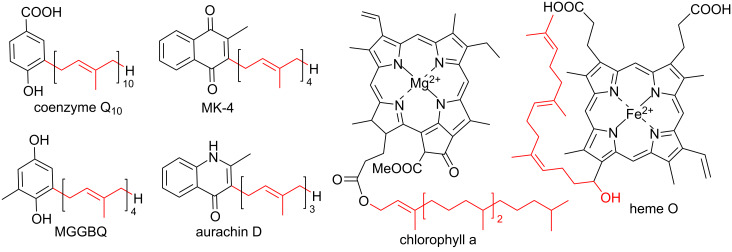
Prenylated aromatic metabolites are involved in cellular processes like cell respiration (coenzyme Q_10_), cell growth and survival (menaquinone MK-4), photosynthesis (chlorophyll a), oxygen reduction (heme O), and biosynthesis of secondary metabolites (MGGBQ: 2-methyl-6-geranylgeranyl-1,4-benzoquinol and aurachin D).

In general, Ptases can be distinguished by their substrate preferences. While the microbial UbiA Ptase catalyzes the C–C-bond formation between an isoprenyl chain and the *meta*-position of *p*-hydroxybenzoate (PHB) in the ubiquinone-Coenzyme Q10 biosynthesis (Figure 1), Ptases of type MenA perform the key step in the menaquinone biosynthesis by prenylating 1,4-dihydroxy-2-naphthoic acid (DHNA) via an intermediate decarboxylative coupling step yielding demethylmenaquinone (DMK) [9]. In contrast, COX10 and chlorophyll synthases are known to fuse prenyl or phytyl tails to porphyrins as acceptors, while homogentisate prenyltransferases catalyze the condensation of homogentisate and geranylgeranyl diphosphate [10–11]. Another intriguing Ptase, called AuaA, has been reported to catalyze the farnesylation of 2-methyl-4-hydroxyquinoline using farnesyl diphosphate (FPP), which results in the metabolite aurachin D [12–13].

Following up on our recent exploration of the biosynthetic repertoire of marine bacteria [14–15], the diversity of the encoded and yet often unexplored bacterial Ptases of Flavobacteria and *Saccharomonospora* strains sparked our interest. In this study we investigated three yet poorly described homologous Ptases within the UbiA superfamily and evaluated their substrate scope by heterologous production and enzymatic bioassays. Results of our study showcase that marine bacteria harbor still a broad unexplored enzymatic repertoire.

## Results and Discussion

### In silico analysis of Ptases in marine Flavobacteria and the genus *Saccharomonospora*

**Figure 2:**
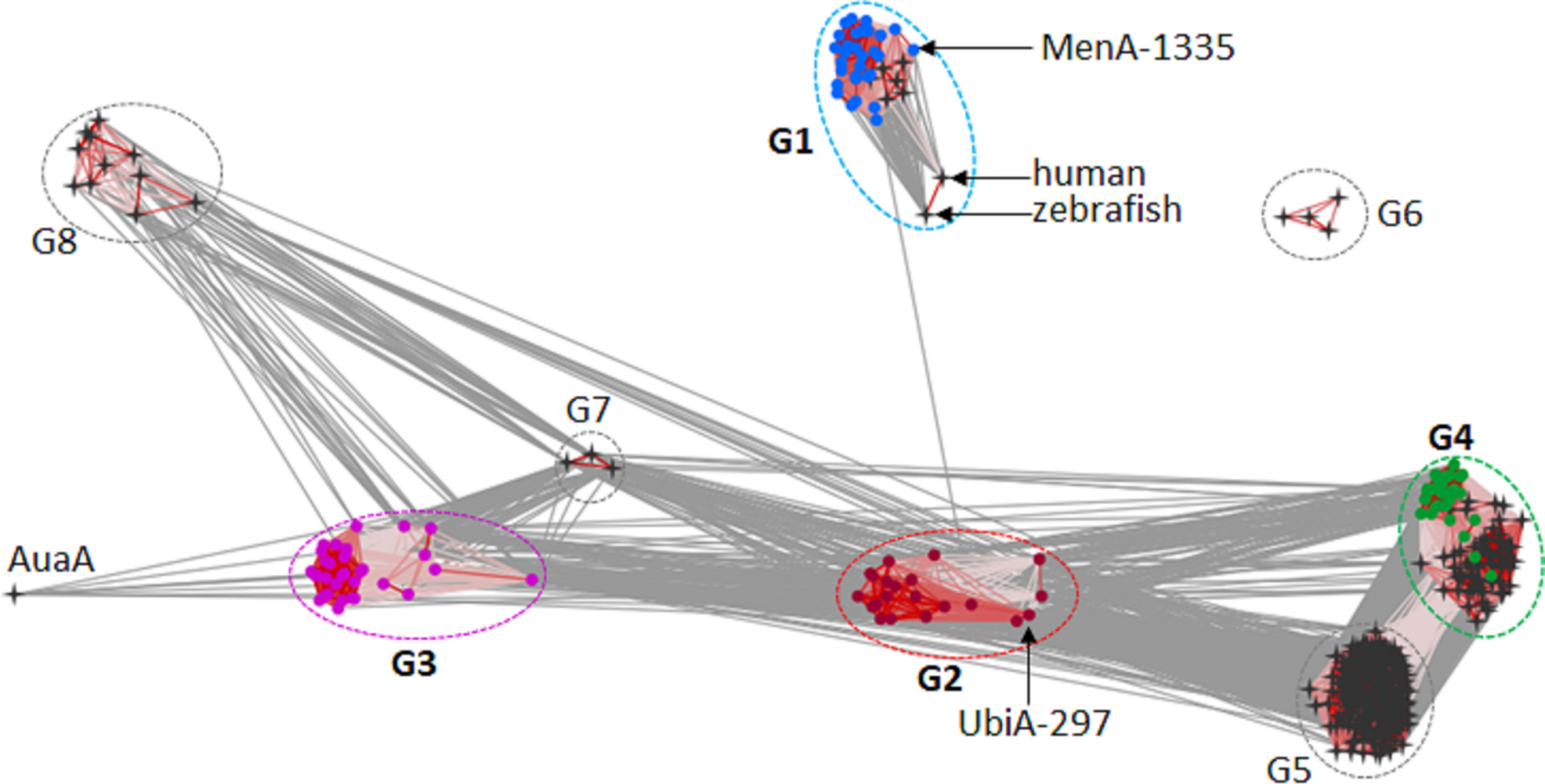
Homology clustering of Ptases encoded in marine *Flavobacteria* and *Saccharomonospora* species (G1–G4, colored nodes) and described Ptases in the Uniprot database (black 4-pointed stars). Visualization of the relationship between proteins is based on their all-against-all pairwise sequence similarities using CLANS. The E-values lower than 1.0E−6 were used to connect each sequence pair by edges. Edges color indicates pairwise identities lower than 10% in grey, 10–45% in light red, and above 45% in dark red. G1: 1,4-dihydroxy-2-naphthoate polyprenyltransferase (blue nodes), G2: UbiA-like Ptases (red nodes), G3: (*S*)-2,3-di-*O*-geranylgeranylglyceryl phosphate synthase (purple nodes), G4: protoheme IX farnesyltransferase (green nodes), G5: 4-hydroxybenzoate octaprenyltransferase (bacterial and mitochondrial), G6: decaprenyl-phosphate phosphoribosyltransferase, G7: chlorophyll synthase, and G8: homogentisate Ptases. AuaA is used as outgroup Ptase from *Stigmatella aurantiaca*.

In a first step, we aimed to gain insights into abundance and diversity of Ptases encoded in both, Flavobacteria and members of the Phylum Actinobacteria, as these were suggested to be involved in the production of meroterpenoids [2]. Thus, the genomes of marine Flavobacteria, including members of the genera *Maribacter*, *Zobellia*, *Algoriphagus*, *Polaribacter*, *Algibacter*, *Arenibacter*, *Echinicola*, *Flavobacterium*, and members of the genus *Saccharomonospora* were subjected to homology searches via local BLAST search. The detected Ptase-related sequences were then subjected to an all-against-all pairwise similarity network with Ptases deposited in the Uniprot database (Figure 2 and Table S1 in Supporting Information File 1). A subsequent network analysis uncovered that the genomes of analyzed bacterial genera encoded only four (G1–G4) out of eight previously reported Ptase groups.

The first group of Ptases (G1) contained close homologs of the bacterial MenA family (EC 2.5.1.74) (50–100% pairwise identity) catalyzing the key step in the menaquinone biosynthesis [2]. As marine bacteria such as *Zobellia barbeyronii* [16] *Marinithermus hydrothermalis* [17], *Marinobacter litoralis* [18], and *Marinobacter flavus* [19] have been reported to produce menaquinone 6 (MK-6), it can be speculated that G1-Ptases of this group are likely involved in its biosynthesis. The second group of Ptases (G2) included yet poorly described UbiA-like Ptases (EC 2.5.1.39) with 43–99% pairwise identity. The third cluster of Ptases (G3) (20–100% pairwise identity) displayed similarities to geranylgeranylglyceryl phosphate synthases (EC 2.5.1.42), which are involved in the formation of polar membrane lipids of archaea and other bacteria [20], while group G4 contained close homologs of the protoheme IX farnesyltransferase (EC 2.5.1.141) (23–100% pairwise identity) responsible for heme O production in bacteria like *E. coli* [21–22]. However, no representatives of the remaining four Ptase groups G6–G8, which encode for ubiquinone biosynthesis (G5), decaprenyl-phosphate phosphoribosyltransferases (EC 2.4.2.45, G6) as reported in Mycobacteriaceae [23], chlorophyll synthases (EC 2.5.1.62, G7) [24] or homogentisate Ptases (EC 2.5.1.117, G8) [25], were found within the investigated genomes of Flavobacteria and *Saccharomonospora* representatives.

A Ptase (UbiA-297) which was putatively assigned as an aromatic Ptase of the G2 UbiA-like family (299 amino acids, calculated mass of 31.9 kDa) (Figure 2) caught our attention due to its low sequence identity on the amino acid sequence level (coverage below 50%) with other characterized Ptases. To further explore the putative function of UbiA-297, we generated a phylogenetic tree using 444 Ptase sequences with already biochemically characterized Ptases; however, none of the members grouped with UbiA-297 the G2-Ptase group (Figure S1, Supporting Information File 1). The closest characterized relatives were identified as a putatively assigned digeranylgeranylglyceryl phosphate synthase encoded in *Sulfurisphaera tokodaii* str. 7 (33% identity) and a 4-hydroxybenzoate octaprenyltransferase (ubiquinone-8 (UQ-8), 23% identity) from *Shewanella woodyi* ATCC 51908. Additionally, UbiA-297 showed sequence identity to a membrane-bound Ptase from the plant *Avena sativa* (31%) [26].

**Figure 3:**
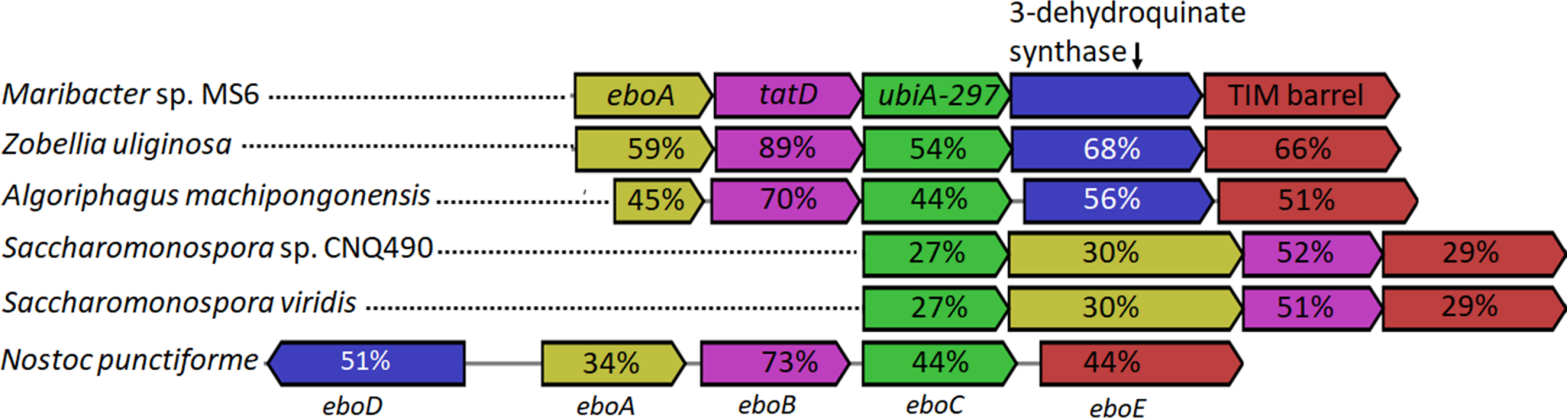
Regional alignment of *ubiA-297* of *Maribacter* sp. MS6 (EU359911.1) and homologous genes in *Z. uliginosa* DSM 2061 (NZ_FTOB01000006), *A. machipongonensis* PR1 (NZ_CM001023), *Saccharomonospora* sp. CNQ490 (NZ_AZUM01000003), *S. viridis* DSM 43017 (NC_013159), and *Nostoc punctiforme* PCC 73102 (NC_010628). Percentage identities of homologous genes are given in the same colored arrow boxes (*Maribacter* sp. MS6 was set as reference).

We then compared the genetic environment of the coding gene *ubiA*-297 (900 bp) within Flavobacteria and *Saccharomonospora* genomes and found a set of conserved genes within the proximity of *ubiA*-297, which were previously identified as *ebo* cluster in bacterial genomes of strains belonging to various different phyla, most notably bacteroidetes and cyanobacteria (Figure 3) [27]. The gene sequence *eboA-E* encodes five yet uncharacterized enzymes, including EboA, a putative metallo-dependent hydrolase TatD (EboB), a putative UbiA prenyltransferase (EboC), a putative 3-dehydroquinate synthase (EboD) likely catalyzing the second step in the shikimate pathway, and a TIM barrel protein (EboE) [28]. While prior studies suggested that the enzymatic reactions carried out by EboA-E include the prenylation of an undetermined substrate by eboC (UbiA-297 homologue) and modifications of a polyhydroxylated aromatic metabolite, the enzymatic reactions carried out by EboA-E have not been investigated yet in detail.

**Figure 4:**
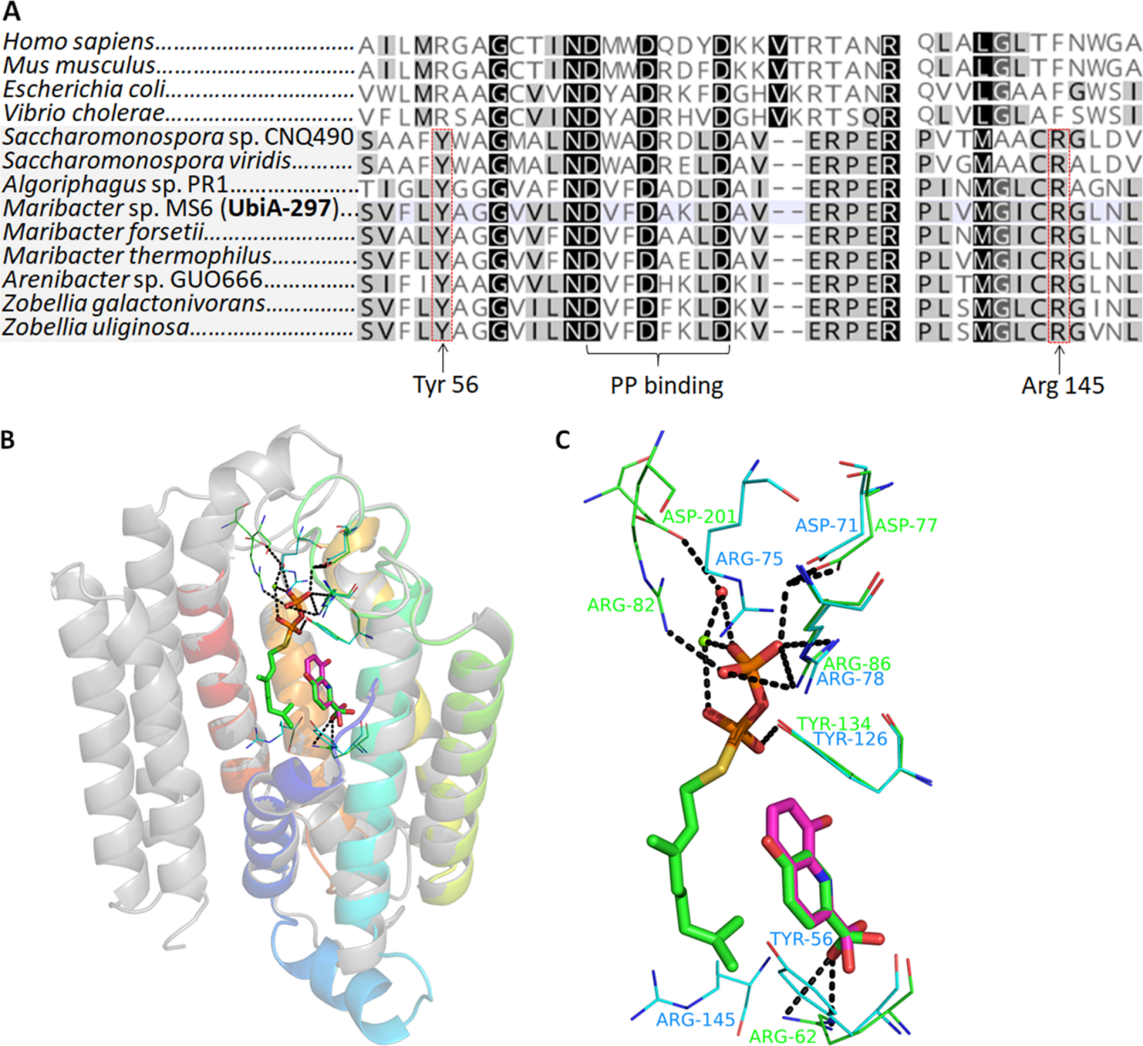
A) Amino acid alignment and binding residues of UbiA-297. G2-Ptases are illustrated in the grey box. B) Homology model of UbiA-297 (in color). Model is based on the substrate-bound structure of a UbiA homolog from *Aeropyrum pernix* sp. K1 (grey). C) The pyrophosphate and aromatic substrate binding sites of UbiA-297 model (shown in green) and template structure (shown in blue). 8-HQA (pink) is paired to 4-HBA (green) after energy minimization.

The wide spread occurrence of the *ebo* gene cluster region within genomes of bacterial symbionts associated, e.g., with marine algae, such as *Maribacter* sp. MS6 [29–31], sparked our interest. To provide more insights into the structural basis of UbiA-297, we performed sequence alignments and structure homology modelling using Swiss-Model (Figure 4) [32], which revealed the transmembrane domain consisting of ten α-helices and loops connecting the transmembrane helices (Figure S2 in Supporting Information File 1), similar to archaeal UbiA prenyltransferase enzymes [33].

The Asp-rich motif of G2-Ptases, known to coordinate Mg^2+^ ions and pyrophosphate, was detectable in all homologous sequences retrieved from marine Flavobacteria and *Saccharomonospora* genomes and was similar to the DXXDXXXD motif in *E. coli* UbiA, but distinct from the motif of other aromatic Ptases such as MenA (DXXDXXXXXD).

While most UbiA Ptases, such as of *E. coli* UbiA (C5A133) and human COQ2 (Q96H96), carry a basic arginine residue within the central cavity and which is proposed to bind the aromatic substrate (e.g., 4-hydroxybenzoate), UbiA-297 and other G2-Ptases harbor instead a tyrosine (Tyr-56) residue, which likely fulfills a similar coordinative function. Furthermore, a conserved arginine residue (UbiA-297 R145) was detectable, which was located in the neighboring α-helix (R145) and in proximity to the binding motif, and was hypothesized to be involved in the coordination process of the aromatic substrate (Figure 4B and 4C).

### Heterologous production of UbiA-297

To enable investigations into the substrate scope of UbiA-297, the coding gene sequence *ubiA-297* was amplified from the genomic DNA of *Maribacter* sp. MS6, while homologous sequences encoded in *Z. uliginosa* DSM 2061 and *A. machipongonensis* sp. PR1 were synthesized. To gain more insights into the binding properties of UbiA-297 and the functional role of the conserved arginine moiety (R145), an additional point-mutated *ubiA-297* (R145A) version was synthesized codon-optimized for expression in *E. coli*. Synthesized and amplified sequences were then cloned into an expression pET28 plasmid containing an N-terminal 6-histidine tag sequence. Heterologous production of enzymes was achieved in *E. coli* BL21 and western blot analysis indicated the accumulation of His-tagged UbiA-297 (35 kD) within concentrated cell membrane fractions (Figure S2, Supporting Information File 1). However, purification of active Ptases failed despite testing different detergent-based purification protocols. Thus, we performed assays with crude protein fractions (1st membrane faction) and protein-enriched membrane fractions, which were obtained after washing and ultracentrifugation.

#### Substrate specificity of UbiA-297

Based on our in silico analysis and previous mass-spectrometry-guided metabolomic analysis of marine Flavobacteria and members of the genus *Saccharomonospora* [29–30], we anticipated hydroxylated aromatic or even quinoline-like acceptor compounds and farnesyl pyrophosphate (FPP) as most likely substrates for Ubi-297 (*Maribacter* sp. MS6). Thus, the 1st membrane faction containing membrane-bound UbiA-297 was subjected to an enzyme assay with farnesyl pyrophosphate (FPP) and different aromatic acceptor substrates in the presence of Mg^2+^ as co-factor. Product formation was monitored after 2 h using high-resolution tandem mass spectrometry (HRMS/MS). Membrane proteins obtained from *E. coli* BL21 cultures harboring the empty expression pET28 plasmid were used as negative control (Figure S3 in Supporting Information File 1).

**Figure 5:**
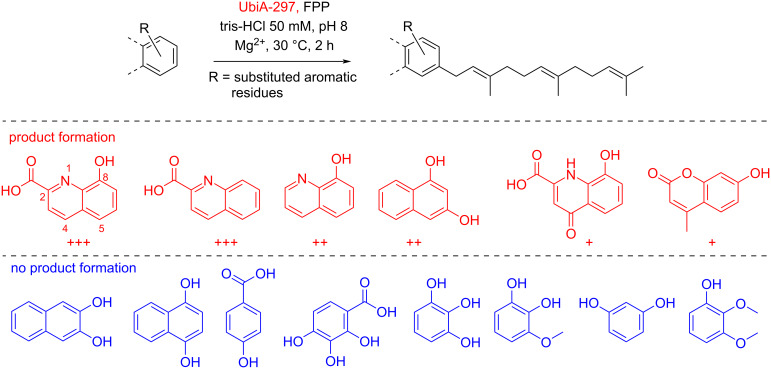
Evaluation of substrate specificity of UbiA-297. Accepted substrates are shown in red, while no product formation was observed for compounds colored in blue. HRMS signal intensities and the MS^2^ pattern of the farnesylated product served as measure to determine preferred acceptor substrates (ion signal intensity: >10^7^ = +++, >10^6^ = ++, and >10^5^ = +, *n* = 3) (for comparision assuming equal ionisation potential).

As depicted in Figure 5, farnesylated products were detectable for six out of 14 tested aromatic acceptor substrates by HRMS/MS (Figures S4–S8 in Supporting Information File 1). In particular, quinoline-type substrates, such as 8-HQA and quinaldic acid, were transformed, while only moderate conversion of 8-hydroxyquinoline and 1,3-dihydroxynaphthalene were observed. Xanthurenic acid and 4-methylumbelliferone were only farnesylated in negligible amounts, and no product formation was observed for phenols or catechols (Figure 5).

**Figure 6:**
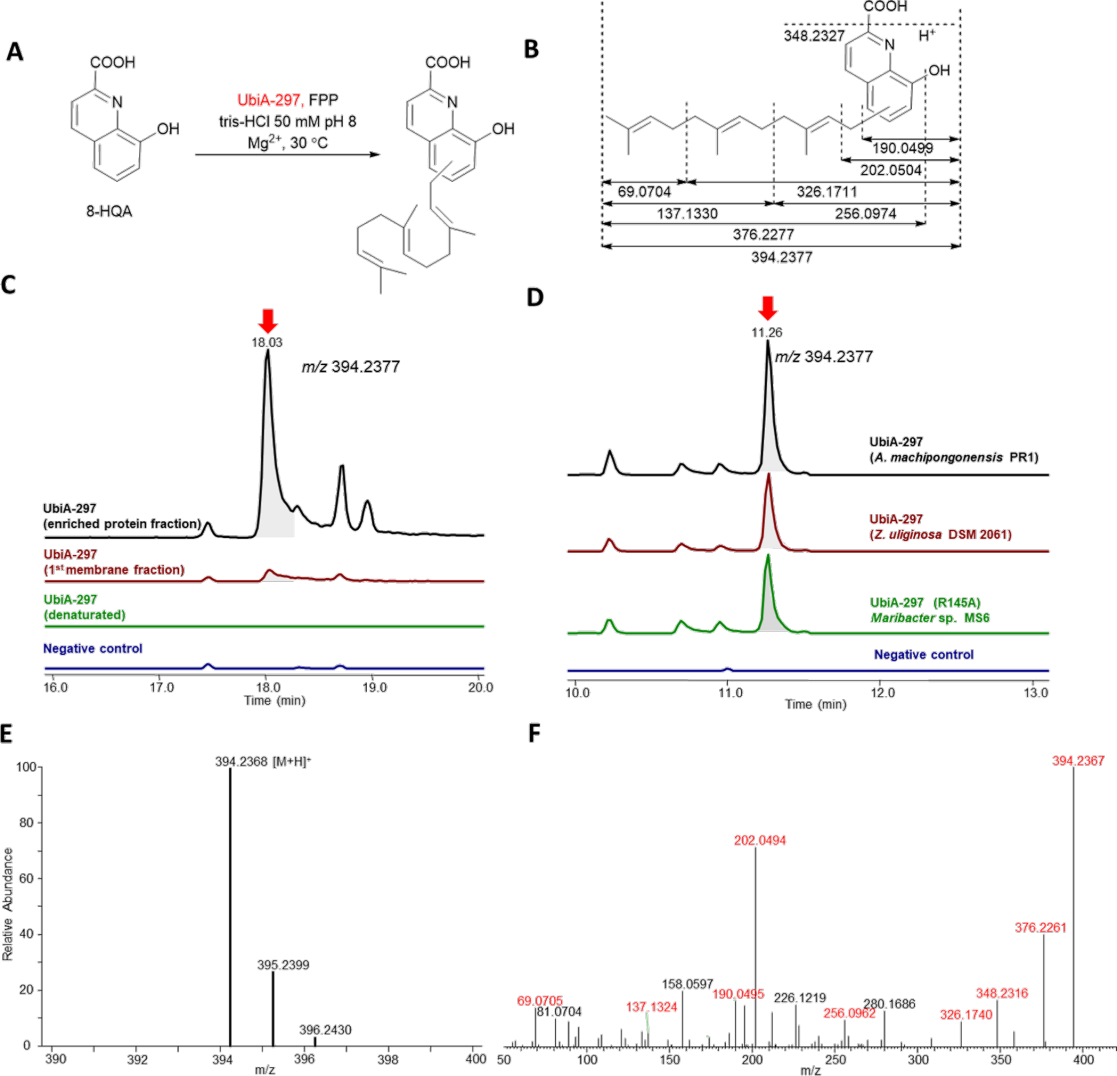
A) Reaction scheme of UbiA-297 catalyzing the assumed *para*-directed farnesylation of 8-HQA; B) calculated MS^2^ fragmentation pattern of farnesylated 8-HQA; C) extracted ion chromatogram (EIC) for *m*/*z* 394.2376 (*t*_R_ 18.03 min, red arrow) of enriched UbiA-297 protein fractions, cell-membrane fraction containing UbiA-297 (*Maribacter* sp. MS6), denaturated enriched UbiA-297 (negative control), and enriched *E. coli* BL21 derived membrane fractions (negative control not shown); fixed ion intensity scale (5.00E7) was applied to all chromatograms; D) EIC for *m*/*z* 394.2376 (*t*_R_ 11.26 min) of assay containing membrane-bound UbiA-297 encoded in *A. machipongonensis* sp. PR1, *Z. uliginosa* DSM 2061, and point mutated UbiA-297 (R145A) from *Maribacter* sp. MS6; E and F) HRMS and MS^2^ spectrum showing fragmentation of prenylated 8-HQA (*m*/*z* 394.2367, *t*_R_ 18.03 min).

Overall, 8-hydroxyquinoline-2-carboxylic acid (8-HQA) appeared to be the most favored substrate amongst the tested panel. Thus, we shortly investigated different reaction parameters using 8-HQA and FPP as substrates. First, we compared the enzyme activity of crude protein fractions directly obtained from cell lysate and enriched UbiA-297 fractions (Figure 6). As expected, higher product signals (RT 18.03 min, *m/z* [M + H]^+^ 394.2368, calcd for C_25_H_32_NO_3_^+^, *m/z* [M + H]^+^ 394.2376; *m/z* [M − H]^−^ 392.2233, calcd for C_25_H_30_NO_3_^−^, [M − H]^−^ 392.2231) were detectable for enriched protein fractions (Figure 6A–C), which suggested that the active protein requires indeed a membrane-like environment. Along these lines, no product formation was detectable when UbiA-297 was denaturated by heating prior to the assay (control), nor when a MenA-1335 (*Maribacter* sp. MS6) homolog was tested.

Furthermore, different pH values and extended incubation times were investigated, however, no significant changes in production levels were noted. Next, enriched protein UbiA-297(R145A), in which the conserved Arg145 (Figure 4A) was replaced by Ala145, was tested (Figure 6D). As similar production levels as UbiA-297 were observed, we concluded that Arg145 might not be crucial for the enzymatic activity.

We then tested, if heterologously produced UbiA-297 homologs from *A. machipongonensis* PR1 and *Z. uliginosa* DSM 2061 (Figure 6D) perform similarly, and indeed observed farnesylation of 8-HQA for both enzymes, which indicated again towards a conserved function of UbiA-297 enzymes within different bacterial taxa.

Furthermore, prenylation activity was also detectable when 8-HQA and farnesol were directly added to an *E. coli* BL21 culture heterologously producing UbiA-297 (Figure S3 in Supporting Information File 1). With the aim to confirm the structure of the prenylated 8-HQA product, a 30 L fermentation of *E. coli*/pET28-297 was performed. After induction of protein expression, the precursor 8-HQA and farnesol were added to the culture and incubated overnight. MS-guided purification of cell lysate and ^1^H NMR analysis of the prenylated 8-HQA product confirmed the prenylation on the quinoline ring (Figure S9 in Supporting Information File 1).

## Conclusion

In silico analysis and homology clustering of Ptases encoded in Flavobacteria and the genus *Sacchromonospora* hinted towards a yet unexplored group of membrane-bound UbiA-like Ptase named UbiA-297, which is part of the conserved *ebo* gene sequences and widespread across various bacterial lineages, including many symbiotic taxa. Heterologously produced UbiA-297 catalyzed the farneslyation of quinoline-like aromatic substrates, with a strong preference for 8-HQA. The herein obtained results build the foundation for future in-depth studies on the substrate scope of Ubi-297-like enzymes and will allow exploring the functions of prenylated 8-HQA for the bacterial producers and their symbionts.

## Experimental

**Chemicals:** Aromatic substrates and the ammonium salt of FPP were obtained from Sigma–Aldrich, Alfa Aesar (Ward Hill, MA), and Acros Organics (Geel, Belgium).

**Bioinformatic analysis:** Amino acid sequences of all described prenyltransferases were retrieved from the UniProtKB/Swiss-Prot database. Other Ptase amino acid sequences of marine Flavobacteria and *Saccharomonopora* strains were obtained using Blast searches against defined genome groups within the PATRIC database (3.6.12) [34]. The sequence clustering was generated by the CLANS (CLuster ANalysis of Sequences) program [35]. In brief, the relationships between sequences were assessed based on an all-against-all BLAST search and the E-values better (lower) than 1.0E−6 were used to connect each sequence pair by edges, which was then colored based on pairwise identities. Different groups were considered based on their distance in space and a combination of E-values and pairwise identities. A phylogenetic tree was created using the neighbor joining method in Geneious Prime (2020.2.3) after multiple sequence alignments using MAFFT (7.450) [36]. Structure modelling of UbiA-297 was done using the SWISS-MODEL server [32] and visualized using Pymol (2.3.3). Regional alignment of homologous Ptase genes was done using MultiGeneBlast [37].

**Nucleotide sequence accession numbers:** The *ubiA-297* and *menA-1335* gene sequences are deposited at GenBank with the accession numbers ON075815 and ON075816, respectively. Accession number or ID of all other sequences used to infer the pairwise similarity network and phylogenetic tree are provided in Supporting Information File 1, Table S1.

**Bacterial strains, plasmids, and culture conditions: ***Maribacter* sp. MS6 was initially isolated from the green macroalga *Ulva mutabilis* [29–30]. The strain was cultivated in 25 mL marine broth (Carl Roth) at 150 rpm and 28 °C for up to four days. For cloning experiments, *E. coli* DH5 alpha and the pJET 1.2 (Thermofisher) and pET28a(+) vectors were used, and *E. coli* BL21 was used as production host. Cultures were supplemented with ampicillin (100 µg mL^−1^) and kanamycin (60 µg mL^−1^) for the selection of plasmids.

**DNA isolation, PCR amplification, and cloning:** gDNA of *Maribacter* sp. MS6 was extracted using DNeasy Blood & Tissue Kit (Qiagen) and PCR amplification of *ubiA*-*297* (922 bp) and *menA-1335* (952 bp) genes was carried out using S7 Fusion High-Fidelity DNA Polymerase (Biozym) and designated primer sequences (*ubiA*-297 forward 5’-CAGGATCCAGGATGTCCAATAAACTAATG-3’ reverse 5’-CGAAGCTTGTCTTAGGTAATGGCAAAAAG-3’; *menA* forward 5’-CAGGATCCCCCTTACTAGTGAC-3’ and reverse 5’-GTAAGCTTGATGGTTGACATTC-3’). The obtained PCR fragments were ligated into a pJET 1.2 vector yielding plasmid pJET-297 and pJET-1335, which were sequenced to confirm integrity. To create the pET28-297 and pET28-1335 expression vector, pJET-297 and pJET-1335 were digested with BamHI and HindIII, and then ligated to digested pET28a(+) with the same restriction enzymes and transformed into *E. coli* BL21 for heterologous expression of UbiA-297 and MenA-1335.

Codon-optimized *ubiA*-*297* homologous genes of *Z. galactanivorans* (DSM 2061) and *A. machipongonensis* (DSM 24695) were synthesized in pET28 vector (BioCAT GmbH) and transformed into *E. coli* BL21 for heterologous expression. Additionally, a modified *ubiA*-*297*(R145A) gene was synthesized and cloned into a pET28 vector (BioCAT GmbH) yielding pET28-297(R145A) for heterologous expression.

**Preparation of enzyme extracts and protein quantification:** Plasmids containing the respective gene were transformed into *E. coli* BL21 and strains harboring the plasmid were cultivated in TB medium supplemented with kanamycin (60 µg mL^−1^). Cultures were grown at 37 °C to an OD_600_ of 0.8, then brought to 16 °C and isopropyl β-ᴅ-thiogalactoside (IPTG) was added to a final concentration of 0.1 mM. Cells were cultivated overnight at 16 °C before harvesting. Cultures were centrifuged, the cell pellet resuspended in Tris-HCl buffer (50 mM, pH 7.8) supplemented with dithiothreitol (DTT, 10 mM), and sonicated on ice (12 min, 100% C, 40% A, 2 seconds on and 3 seconds off intervals using Hielscher UP200St ultrasonic processor). The first cell membrane fraction was obtained by centrifugation (12000*g*, 20 min, 4 °C), while the enriched protein fraction, likely imbedded in lipid rafts, required ultracentrifugation of the crude protein lysates (240000*g*, 90 min). The obtained protein pellet was resuspended in the same buffer as used for the bioassays. Protein concentration was measured according to Bradford [38].

**Enzyme assays:** All the enzyme assays were performed using a standard reaction mixture (100 µL) by adding 50 µL Tris-HCl (50 mM, pH 7.8) containing MgCl_2_, FPP, aromatic substrate (each 1 mM), and 50 µL of membrane-bound protein aliquots with a total protein content of 0.4 mg. The reaction mixtures were incubated at 30 °C for 2 h (extended period of time) and extracted subsequently three times with ethyl acetate (450 µL). The solvent was removed in vacuo, the residue was dissolved in methanol (100 µL), and analyzed by HRMS/MS. Denaturation of proteins was performed at 95 °C for 10 min (negative control). Additionally, *E. coli* BL21 derived enriched membrane fractions served as negative control.

**In vivo assays:** In vivo assays (100 mL) were performed using *E. coli* BL21 harboring one of the following vectors: pET28-297, pET28-1335, and pET28-no insert. Protein expression was performed under the same conditions explained above, and after 12 h 8-HQA, farnesol, and MgCl_2_ were added to each culture to a final concentration of 1 mM each and cultivation was continued at 30 °C (3 d). Subsequently, cultures were extracted twice with ethyl acetate (400 mL), the solvent was removed in vacuo*,* the residue redissolved in MeOH, and subjected to HRMS/MS analysis.

**Fermentation and purification:** Fermentation of *E. coli* BL21 cells harboring the pET28-297 vector was performed in a 75 L X-Cube Bioreactor (Braun Biotech International) using 30 L of Terrific Broth medium (Carl Roth GmbH) enriched with 4 mL/L glycerol and 60 mg/L kanamycin. Similar to the in vivo culture assays, the culture was cooled to 16 °C at an OD_600_ of 1 and heterologous production induced with IPTG (final concentration of 0.1 mM). After 12 h, 8-HQA, farnesol, and MgCl_2_ were added to the fermentation with a final concentration of 1 mM. Fermentation was continued for 3 d at 30 °C, after which the culture was centrifuged and the cell biomass was lyophilized. Dry cell mass (103.6 g) was extracted with methanol (1.0 L), dried under vacuum, and the resultant MeOH extract was then extracted with hexane (120 mL) and dried again under vacuum. The hexane extract was purified by flash chromatography (Biotage Isolera Prime) over a silica gel column (eluent: cyclohexane/EtOAc 100:0 to 80:20 to 0:100). The appropriate fraction was collected, evaporated, and purified by preparative HPLC (Shimadzu) over a phenyl-hexyl column (Luna, 5 µm, 250 × 21.2 mm, 100 Å) (eluent: H_2_O/MeCN + 0.1% formic acid 80:20 to 50:50). The appropriate fraction was collected and evaporated to afford farnesylated 8-HQA.

**HRMS/MS analysis of the enzymatic products:** UHPLC-HESI-HRMS measurement was performed on a Dionex Ultimate3000 system combined with a Q-Exactive Plus mass spectrometer (Thermo Scientific) with a heated electrospray ion source (HESI). Metabolite separation was carried out by reversed-phase liquid chromatography at 40 °C using a Luna Omega C18 column (100 × 2.1 mm, 1.6 μm, 100 Å, Phenomenex) preceded by a SecurityGuardTM ULTRA guard cartridge (2 × 2.1 mm, Phenomenex). Mobile phases were acidified with 0.1% formic acid and consisted of H_2_O (A), and acetonitrile (B) with a flow rate of 0.3 mL/min and the injection volume was 5 µL. The prenylated products were separated under either long or short gradient runs. The long gradient run was 30 min as follows: 0–0.5 min, 5% B; 0.5–18 min, 5–97% B; 18–25 min, 97–5% B; 25–30 min, 5% B. The short gradient run was 15 min as follows: 0–0.8 min, 40% B; 0.8–10 min, 40–97% B; 10–12 min, 97% B; 12–13 min, 40% B; 13–15 min, 40% B. The retention times of the farnesylated 8HQA was 18.03 min using a long gradient run and 11.26 min using a short gradient run.

**Spectroscopic analysis of the enzyme products: **^1^H NMR spectra was carried out using a Bruker AVANCE III 600 MHz spectrometer equipped with a Bruker Cryoplatform with chemical shifts given in ppm (δ).

## Supporting Information

File 1 Sequence analysis and copies of MS/MS and NMR spectra.
