# Supplementary material for: Identification of the new prenyltransferase Ubi-297 from marine bacteria and elucidation of its substrate specificity
Source: Beilstein J Org Chem. 2022 Jun 22;18:722–31. doi: 10.3762/bjoc.18.72 (PMC9235831; doi:10.3762/bjoc.18.72)
Supplement: File 1 — Sequence analysis and copies of MS/MS and NMR spectra. [file Beilstein_J_Org_Chem-18-722-s001.pdf]

BEILSTEIN JOURNAL OF ORGANIC CHEMISTRY

Supporting Information

for

Identification of the new prenyltransferase Ubi-297 from marine bacteria and elucidation of its substrate specificity

Jamshid Amiri Moghaddam, Huijuan Guo, Karsten Willing, Thomas Wichard and Christine Beemelmanns

Beilstein J. Org. Chem. **2022,** *18,* 722–731. doi:10.3762/bjoc.18.72

Sequence analysis and copies of MS/MS and NMR spectra

License and Terms: This is a supporting information file under the terms of the Creative Commons Attribution License (https://creativecommons.org/licenses/by/4.0). Please note that the reuse, redistribution and reproduction in particular requires that the author(s) and source are credited and that individual graphics may be subject to special legal provisions.
The license is subject to the *Beilstein Journal of Organic Chemistry* terms and conditions: (https://www.beilstein-journals.org/bjoc/terms)

Table of contents

Table S1: List of all Ptases used in all-against-all pairwise sequence similarity network and phylogenetic tree S3

Figure S1: Phylogenetic tree of Ptases from marine Flavobacteria, Sacharomonospora and Uniprot database. G1: 1,4-dihydroxy-2-naphthoate polyprenyltransferase (blue), G2: UbiA-like PTases (red), G3: (S)-2,3-di-O-geranylgeranylglyceryl phosphate synthase (purple), G4: Protoheme IX farnesyltransferase (green). Phylogenetic tree was constructed using clustal omega multiple alignment and neighbor-joining method. 500 replicates were used for bootstrap resampling method S15

Figure S2: A) Heterologous expression of G2 UbiA-297 of *Maribacter* sp. MS6 in E. coli. Lanes from left to right: Crude lysate (L), membrane fraction (MF), PD10 column flow through (PD10), NiNTA flow through (FT), NiNTA elution1- 100 mM imidazol (E1), NiNTA elution2- 150 mM imidazol (E2), NiNTA elution3- 200 mM imidazol (E3), NiNTA elution4- 300 mM imidazol (E4), Biorad all blue protein marker (M) and size indication of marker in kD. B) TMHMM posterior probabilities for G2 UbiA-297 of *Maribacter* sp. MS6. S16

Figure S3: Extracted Ion Chromatogram (EIC for m/z 378.2428) for the in vivo assay extracts of UbiA-297 using 8-HQA and FPP as substrates and HR-MS of product peak at RT 18.03 min S17

Figure S4: HRMS/MS analysis of the in vitro assays using quinaldic acid and FPP as substrates (short gradient, 15 min run). A) Extracted Ion Chromatogram (EIC for m/z 378.2428) for the assay extracts of UbiA-297 membrane fraction, crude protein lysate, and denaturated membrane fraction. Fixed ion intensity scale (1.05E7) was applied to all chromatograms; B) MS-spectrum of the peak at 11.10 min from the membrane fraction assay of UbiA-297; C) MS/MS fragmentation of prenylated product at m/z 378.2424. (Putative fragmentation pattern of farnesylated quinaldic acid is depicted) S18

Figure S5: HRMS/MS analysis of the in vitro assays using 8-hydroxyquinoline and FPP as substrates (short gradient, 15 min run). A) Extracted Ion Chromatogram (EIC for m/z 350.2479) for the assay extracts of UbiA-297 membrane fraction, crude protein lysate, and denaturated membrane fraction. Fixed ion intensity scale (2.82E6) was applied to all chromatograms; B) MS-spectrum of the peak at 11.10 min from the membrane fraction assay of UbiA-297; C) MS/MS fragmentation of prenylated product at m/z 350.2479. (Putative fragmentation pattern of farnesylated 8-hydroxyquinoline is depicted). S19

Figure S6: HRMS/MS analysis of the in vitro assays using 1,3-dihydroxynaphtalene and FPP as substrates (short gradient,15 min run). A) Extracted Ion Chromatogram (EIC for m/z 365.2476) for the assay extracts of UbiA-297 membrane fraction, crude protein lysate, and denaturated membrane fraction. Fixed ion intensity scale (2.05E6) was applied to all chromatograms; B) MS-spectrum of the peak at 11.10 min from the membrane fraction assay of UbiA-297; C) MS/MS fragmentation of prenylated product at m/z 365.2474. (Putative fragmentation pattern of farnesylated 1,3-dihydroxynaphtalene is depicted) S20

Figure S7: HRMS/MS analysis of the in vitro assays using 4-methylumbelliferone and FPP as substrates (short gradient,15 min run). A) Extracted Ion Chromatogram (EIC for m/z 381.2425) for the assay extracts of UbiA-297 membrane fraction, crude protein lysate, and denaturated membrane fraction. Fixed ion intensity scale (7.45E5) was applied to all chromatograms; B) MS-spectrum of the peak at 11.10 min from the membrane fraction assay of UbiA-297; C) MS/MS fragmentation of prenylated product at m/z 381.2425. (Putative fragmentation pattern of farnesylated 4-methylumbelliferone is depicted) S21

Figure S8: HRMS/MS analysis of the in vitro assays using xanthurenic acid and FPP as substrates (short gradient, 15 min run). A) Extracted Ion Chromatogram (EIC for m/z 410.2326) for the assay extracts of UbiA-297 membrane fraction, crude protein lysate, and denaturated membrane fraction. Fixed ion intensity scale (3.14E5) was applied to all chromatograms; B) MS-spectrum of the peak at 11.10 min from the membrane fraction assay of UbiA-297 S22

Figure S9: Overlayed ^{1}H NMR spectra of A) isolated product with m/z 394.2376 (blue), and B) starting material 8-HQA (red), 600 Mhz (d5-pyridine). S23

Table S1: List of all Ptases used in all-against-all pairwise sequence similarity network and phylogenetic tree

Nr	Accession/ ID*	Gene Product
1	1122191.4.peg.195	Heme O synthase, protoheme IX farnesyltransferase, COX10-CtaB [*Maribacter antarcticus* DSM 21422
2	1121859.3.peg.1165	Heme O synthase, protoheme IX farnesyltransferase, COX10-CtaB [*Echinicola pacifica* DSM 19836
3	1121859.3.peg.1481	1,4-dihydroxy-2-naphthoate polyprenyltransferase (EC 2.5.1.74) [*Echinicola pacifica* DSM 19836
4	1121859.3.peg.361	Similar to (S)-2,3-di-O-geranylgeranylglyceryl phosphate synthase [*Echinicola pacifica* DSM 19836
5	1122191.4.peg.1189	Aromatic prenyltransferase 1, UbiA family [*Maribacter antarcticus* DSM 21422
6	1122191.4.peg.2126	1,4-dihydroxy-2-naphthoate polyprenyltransferase (EC 2.5.1.74) [*Maribacter antarcticus* DSM 21422
7	1122191.4.peg.3274	Similar to (S)-2,3-di-O-geranylgeranylglyceryl phosphate synthase [*Maribacter antarcticus* DSM 21422
8	1137271.3.peg.647	Heme O synthase, protoheme IX farnesyltransferase, COX10-CtaB [*Saccharomonospora* sp. CNQ490
9	1137271.3.peg.2500	Aromatic prenyltransferase 1, UbiA family [*Saccharomonospora* sp. CNQ490
10	1197874.4.peg.1286	1,4-dihydroxy-2-naphthoate polyprenyltransferase (EC 2.5.1.74) [*Maribacter thermophilus* strain HT7-2
11	1197874.4.peg.2089	Heme O synthase, protoheme IX farnesyltransferase, COX10-CtaB [*Maribacter thermophilus* strain HT7-2
12	1197874.4.peg.222	Aromatic prenyltransferase 1, UbiA family [*Maribacter thermophilus* strain HT7-2
13	1197874.4.peg.3133	Similar to (S)-2,3-di-O-geranylgeranylglyceryl phosphate synthase [*Maribacter thermophilus* strain HT7-2
14	1137271.3.peg.2913	1,4-dihydroxy-2-naphthoate polyprenyltransferase (EC 2.5.1.74) [*Saccharomonospora* sp. CNQ490
15	1206753.3.peg.805	Heme O synthase, protoheme IX farnesyltransferase, COX10-CtaB [*Maribacter spongiicola* strain DSM 25233
16	1206753.3.peg.2854	Similar to (S)-2,3-di-O-geranylgeranylglyceryl phosphate synthase [*Maribacter spongiicola* strain DSM 25233
17	1206753.3.peg.1779	1,4-dihydroxy-2-naphthoate polyprenyltransferase (EC 2.5.1.74) [*Maribacter spongiicola* strain DSM 25233
18	1206753.3.peg.3728	Aromatic prenyltransferase 1, UbiA family [*Maribacter spongiicola* strain DSM 25233
19	1206816.3.peg.1205	Similar to (S)-2,3-di-O-geranylgeranylglyceryl phosphate synthase [*Maribacter vaceletii* strain DSM 25230
20	1206816.3.peg.161	Heme O synthase, protoheme IX farnesyltransferase, COX10-CtaB [*Maribacter vaceletii* strain DSM 25230
21	1206816.3.peg.1605	1,4-dihydroxy-2-naphthoate polyprenyltransferase (EC 2.5.1.74) [*Maribacter vaceletii* strain DSM 25230
22	1236486.3.peg.383	1,4-dihydroxy-2-naphthoate polyprenyltransferase (EC 2.5.1.74) [*Flavobacterium aquaticum* strain CGMCC 1.12398
23	1236486.3.peg.1831	Similar to (S)-2,3-di-O-geranylgeranylglyceryl phosphate synthase [*Flavobacterium aquaticum* strain CGMCC 1.12398
24	1236486.3.peg.771	Heme O synthase, protoheme IX farnesyltransferase, COX10-CtaB [*Flavobacterium aquaticum* strain CGMCC 1.12398
25	1236486.3.peg.2092	Similar to (S)-2,3-di-O-geranylgeranylglyceryl phosphate synthase [*Flavobacterium aquaticum* strain CGMCC 1.12398
26	1236486.7.peg.991	Similar to (S)-2,3-di-O-geranylgeranylglyceryl phosphate synthase [*Flavobacterium aquaticum* strain 1Y8A
27	1236486.7.peg.355	Heme O synthase, protoheme IX farnesyltransferase, COX10-CtaB [*Flavobacterium aquaticum* strain 1Y8A
28	1236486.7.peg.1767	1,4-dihydroxy-2-naphthoate polyprenyltransferase (EC 2.5.1.74) [*Flavobacterium aquaticum* strain 1Y8A
29	1236486.7.peg.2298	Similar to (S)-2,3-di-O-geranylgeranylglyceryl phosphate synthase [*Flavobacterium aquaticum* strain 1Y8A
30	1392498.4.peg.1577	1,4-dihydroxy-2-naphthoate polyprenyltransferase (EC 2.5.1.74) [*Maribacter forsetii* DSM 18668
31	1392498.4.peg.2850	Aromatic prenyltransferase 1, UbiA family [*Maribacter forsetii* DSM 18668
32	1392498.4.peg.3593	Similar to (S)-2,3-di-O-geranylgeranylglyceryl phosphate synthase [*Maribacter forsetii* DSM 18668
33	1392498.4.peg.742	Heme O synthase, protoheme IX farnesyltransferase, COX10-CtaB [*Maribacter forsetii* DSM 18668
34	143224.4.peg.2004	Aromatic prenyltransferase 1, UbiA family [*Zobellia uliginosa* strain MAR_2009_138
35	143224.4.peg.1185	1,4-dihydroxy-2-naphthoate polyprenyltransferase (EC 2.5.1.74) [*Zobellia uliginosa* strain MAR_2009_138
36	143224.4.peg.2755	Similar to (S)-2,3-di-O-geranylgeranylglyceryl phosphate synthase [*Zobellia uliginosa* strain MAR_2009_138
37	143224.4.peg.3880	Heme O synthase, protoheme IX farnesyltransferase, COX10-CtaB [*Zobellia uliginosa* strain MAR_2009_138

38	143224.8.peg.3999	Similar to (S)-2,3-di-O-geranylgeranylglyceryl phosphate synthase [*Zobellia uliginosa* strain DSM 2061
39	143224.8.peg.3051	1,4-dihydroxy-2-naphthoate polyprenyltransferase (EC 2.5.1.74) [*Zobellia uliginosa* strain DSM 2061
40	143224.8.peg.265	Heme O synthase, protoheme IX farnesyltransferase, COX10-CtaB [*Zobellia uliginosa* strain DSM 2061
41	143224.8.peg.1269	Aromatic prenyltransferase 1, UbiA family [*Zobellia uliginosa* strain DSM 2061
42	1608400.3.peg.849	Similar to (S)-2,3-di-O-geranylgeranylglyceryl phosphate synthase [*Algibacter amylolyticus* strain RU-4-M-4
43	1608400.3.peg.587	Heme O synthase, protoheme IX farnesyltransferase, COX10-CtaB [*Algibacter amylolyticus* strain RU-4-M-4
44	1608400.3.peg.2023	1,4-dihydroxy-2-naphthoate polyprenyltransferase (EC 2.5.1.74) [*Algibacter amylolyticus* strain RU-4-M-4
45	1608400.3.peg.3167	Similar to (S)-2,3-di-O-geranylgeranylglyceryl phosphate synthase [*Algibacter amylolyticus* strain RU-4-M-4
46	1608400.5.peg.850	Similar to (S)-2,3-di-O-geranylgeranylglyceryl phosphate synthase [*Algibacter amylolyticus* strain DSM 29199
47	1608400.5.peg.589	Heme O synthase, protoheme IX farnesyltransferase, COX10-CtaB [*Algibacter amylolyticus* strain DSM 29199
48	1608400.5.peg.1276	Similar to (S)-2,3-di-O-geranylgeranylglyceryl phosphate synthase [*Algibacter amylolyticus* strain DSM 29199
49	1608400.5.peg.1668	1,4-dihydroxy-2-naphthoate polyprenyltransferase (EC 2.5.1.74) [*Algibacter amylolyticus* strain DSM 29199
50	1658664.4.peg.1849	Similar to (S)-2,3-di-O-geranylgeranylglyceryl phosphate synthase [*Maribacter flavus* strain C3 strain KCTC 42508
51	1658664.4.peg.3317	1,4-dihydroxy-2-naphthoate polyprenyltransferase (EC 2.5.1.74) [*Maribacter flavus* strain C3 strain KCTC 42508
52	1658664.4.peg.617	Heme O synthase, protoheme IX farnesyltransferase, COX10-CtaB [*Maribacter flavus* strain C3 strain KCTC 42508
53	1658664.4.peg.2331	Aromatic prenyltransferase 1, UbiA family [*Maribacter flavus* strain C3 strain KCTC 42508
54	1775173.3.peg.1572	hypothetical protein [*Polaribacter pacificus* strain CGMCC 1.15763
55	1775173.3.peg.1367	Similar to (S)-2,3-di-O-geranylgeranylglyceryl phosphate synthase [*Polaribacter pacificus* strain CGMCC 1.15763
56	1775173.3.peg.988	Heme O synthase, protoheme IX farnesyltransferase, COX10-CtaB [*Polaribacter pacificus* strain CGMCC 1.15763
57	1775173.3.peg.1950	1,4-dihydroxy-2-naphthoate polyprenyltransferase (EC 2.5.1.74) [*Polaribacter pacificus* strain CGMCC 1.15763
58	228956.4.peg.716	Heme O synthase, protoheme IX farnesyltransferase, COX10-CtaB [*Maribacter sedimenticola* strain DSM 19840
59	228956.4.peg.1171	Aromatic prenyltransferase 1, UbiA family [*Maribacter sedimenticola* strain DSM 19840
60	228956.4.peg.2235	Similar to (S)-2,3-di-O-geranylgeranylglyceryl phosphate synthase [*Maribacter sedimenticola* strain DSM 19840
61	228956.4.peg.2939	1,4-dihydroxy-2-naphthoate polyprenyltransferase (EC 2.5.1.74) [*Maribacter sedimenticola* strain DSM 19840
62	228957.5.peg.2049	Similar to (S)-2,3-di-O-geranylgeranylglyceryl phosphate synthase [*Maribacter orientalis* strain DSM 16471
63	228957.5.peg.2580	1,4-dihydroxy-2-naphthoate polyprenyltransferase (EC 2.5.1.74) [*Maribacter orientalis* strain DSM 16471
64	228957.5.peg.651	Heme O synthase, protoheme IX farnesyltransferase, COX10-CtaB [*Maribacter orientalis* strain DSM 16471
65	228957.5.peg.246	Aromatic prenyltransferase 1, UbiA family [*Maribacter orientalis* strain DSM 16471
66	228958.3.peg.335	Similar to (S)-2,3-di-O-geranylgeranylglyceryl phosphate synthase [*Maribacter aquivivus* strain DSM 16478
67	228958.3.peg.1364	Heme O synthase, protoheme IX farnesyltransferase, COX10-CtaB [*Maribacter aquivivus* strain DSM 16478
68	228958.3.peg.3570	Aromatic prenyltransferase 1, UbiA family [*Maribacter aquivivus* strain DSM 16478
69	228958.3.peg.2310	1,4-dihydroxy-2-naphthoate polyprenyltransferase (EC 2.5.1.74) [*Maribacter aquivivus* strain DSM 16478
70	228959.3.peg.1400	Heme O synthase, protoheme IX farnesyltransferase, COX10-CtaB *[Maribacter ulvicola* strain DSM 15366
71	228959.3.peg.271	Similar to (S)-2,3-di-O-geranylgeranylglyceryl phosphate synthase *[Maribacter ulvicola* strain DSM 15366
72	228959.3.peg.2970	1,4-dihydroxy-2-naphthoate polyprenyltransferase (EC 2.5.1.74) *[Maribacter ulvicola* strain DSM 15366
73	228959.3.peg.2320	Aromatic prenyltransferase 1, UbiA family *[Maribacter ulvicola* strain DSM 15366
74	2489054.3.peg.102	Heme O synthase, protoheme IX farnesyltransferase, COX10-CtaB [*Arenibacter* sp. GUO666
75	2489054.3.peg.1062	Similar to (S)-2,3-di-O-geranylgeranylglyceryl phosphate synthase [*Arenibacter* sp. GUO666
76	2489054.3.peg.2793	Aromatic prenyltransferase 1, UbiA family [*Arenibacter* sp. GUO666
77	2489054.3.peg.3362	1,4-dihydroxy-2-naphthoate polyprenyltransferase (EC 2.5.1.74) [*Arenibacter* sp. GUO666

78	252356.13.peg.1335	1,4-dihydroxy-2-naphthoate polyprenyltransferase (EC 2.5.1.74) [*Maribacter* MS6
79	252356.13.peg.2206	Heme O synthase, protoheme IX farnesyltransferase, COX10-CtaB [*Maribacter* MS6
80	252356.13.peg.297	Aromatic prenyltransferase 1, UbiA family [*Maribacter* MS6
81	313603.6.peg.1920	Similar to (S)-2,3-di-O-geranylgeranylglyceryl phosphate synthase [*Maribacter* sp. HTCC2170
82	252356.13.peg.3531	Similar to (S)-2,3-di-O-geranylgeranylglyceryl phosphate synthase [*Maribacter* MS6
83	313603.6.peg.2860	1,4-dihydroxy-2-naphthoate polyprenyltransferase (EC 2.5.1.74) [*Maribacter* sp. HTCC2170
84	313603.6.peg.867	Heme O synthase, protoheme IX farnesyltransferase, COX10-CtaB [*Maribacter* sp. HTCC2170
85	320912.15.peg.1774	Heme O synthase, protoheme IX farnesyltransferase, COX10-CtaB [*Maribacter dokdonensis* strain MAR_2009_71
86	320912.15.peg.60	Aromatic prenyltransferase 1, UbiA family [*Maribacter dokdonensis* strain MAR_2009_71
87	320912.15.peg.2606	1,4-dihydroxy-2-naphthoate polyprenyltransferase (EC 2.5.1.74) [*Maribacter dokdonensis* strain MAR_2009_71
88	320912.15.peg.709	Similar to (S)-2,3-di-O-geranylgeranylglyceryl phosphate synthase [*Maribacter dokdonensis* strain MAR_2009_71
89	388413.5.peg.816	1,4-dihydroxy-2-naphthoate polyprenyltransferase (EC 2.5.1.74) [Algoriphagus sp. PR1
90	388413.5.peg.2653	Aromatic prenyltransferase 1, UbiA family [Algoriphagus sp. PR1
91	388413.5.peg.2670	Similar to (S)-2,3-di-O-geranylgeranylglyceryl phosphate synthase [Algoriphagus sp. PR1
92	388413.5.peg.2771	Heme O synthase, protoheme IX farnesyltransferase, COX10-CtaB [Algoriphagus sp. PR1
93	429344.6.peg.730	Similar to (S)-2,3-di-O-geranylgeranylglyceryl phosphate synthase [*Maribacter* polysiphoniae strain DSM 23514
94	429344.6.peg.2764	Heme O synthase, protoheme IX farnesyltransferase, COX10-CtaB [*Maribacter* polysiphoniae strain DSM 23514
95	429344.6.peg.3072	1,4-dihydroxy-2-naphthoate polyprenyltransferase (EC 2.5.1.74) [*Maribacter* polysiphoniae strain DSM 23514
96	440514.5.peg.3165	Heme O synthase, protoheme IX farnesyltransferase, COX10-CtaB [*Maribacter* stanieri strain DSM 19891
97	440514.5.peg.2094	Similar to (S)-2,3-di-O-geranylgeranylglyceryl phosphate synthase [*Maribacter* stanieri strain DSM 19891
98	440514.5.peg.245	Aromatic prenyltransferase 1, UbiA family [*Maribacter* stanieri strain DSM 19891
99	440514.5.peg.427	1,4-dihydroxy-2-naphthoate polyprenyltransferase (EC 2.5.1.74) [*Maribacter* stanieri strain DSM 19891
100	471857.5.peg.1109	Aromatic prenyltransferase 1, UbiA family [*Saccharomonospora* viridis DSM 43017
101	471857.5.peg.1596	Heme O synthase, protoheme IX farnesyltransferase, COX10-CtaB [*Saccharomonospora* viridis DSM 43017
102	561365.3.peg.1410	Similar to (S)-2,3-di-O-geranylgeranylglyceryl phosphate synthase [*Maribacter* arcticus strain DSM 23546
103	471857.5.peg.302	1,4-dihydroxy-2-naphthoate polyprenyltransferase (EC 2.5.1.74) [*Saccharomonospora* viridis DSM 43017
104	561365.3.peg.1994	Heme O synthase, protoheme IX farnesyltransferase, COX10-CtaB [*Maribacter* arcticus strain DSM 23546
105	561365.3.peg.1178	1,4-dihydroxy-2-naphthoate polyprenyltransferase (EC 2.5.1.74) [*Maribacter* arcticus strain DSM 23546
106	561365.3.peg.2786	Aromatic prenyltransferase 1, UbiA family [*Maribacter* arcticus strain DSM 23546
107	63186.21.peg.2087	Heme O synthase, protoheme IX farnesyltransferase, COX10-CtaB [*Zobellia galactanivorans* strain OII3
108	63186.21.peg.896	Similar to (S)-2,3-di-O-geranylgeranylglyceryl phosphate synthase [*Zobellia galactanivorans* strain OII3
109	63186.21.peg.2425	1,4-dihydroxy-2-naphthoate polyprenyltransferase (EC 2.5.1.74) [*Zobellia galactanivorans* strain OII3
110	63186.21.peg.4811	Aromatic prenyltransferase 1, UbiA family [*Zobellia galactanivorans* strain OII3
111	63186.3.peg.1224	Similar to (S)-2,3-di-O-geranylgeranylglyceryl phosphate synthase [*Zobellia galactanivorans* strain Dsij
112	63186.3.peg.2343	Heme O synthase, protoheme IX farnesyltransferase, COX10-CtaB [*Zobellia galactanivorans* strain Dsij
113	63186.3.peg.489	Aromatic prenyltransferase 1, UbiA family [*Zobellia galactanivorans* strain Dsij
114	63186.3.peg.3937	1,4-dihydroxy-2-naphthoate polyprenyltransferase (EC 2.5.1.74) [*Zobellia galactanivorans* strain Dsij
115	B1KKU2	UBIA_SHEWM 4-hydroxybenzoate octaprenyltransferase OS=*Shewanella woodyi* (strain ATCC 51908 / MS32) OX=392500 GN=ubiA PE=3 SV=1
116	B0TRE7	UBIA_SHEHH 4-hydroxybenzoate octaprenyltransferase OS=*Shewanella halifaxensis* (strain HAW-EB4) OX=458817 GN=ubiA PE=3 SV=1
117	Q66FH0	UBIA_YERPS 4-hydroxybenzoate octaprenyltransferase OS=*Yersinia pseudotuberculosis* serotype I (strain IP32953) OX=273123 GN=ubiA PE=3 SV=1

118	B2SXA0	UBIA_PARPJ 4-hydroxybenzoate octaprenyltransferase OS=*Paraburkholderia phytofirmans* (strain DSM 17436 / LMG 22146 / PsJN) OX=398527 GN=ubiA PE=3 SV=1
119	Q1C0T8	UBIA_YERPA 4-hydroxybenzoate octaprenyltransferase OS=*Yersinia pestis* bv. Antiqua (strain Antiqua) OX=360102 GN=ubiA PE=3 SV=1
120	Q9PH76	UBIA_XYLFA 4-hydroxybenzoate octaprenyltransferase OS=*Xylella fastidiosa* (strain 9a5c) OX=160492 GN=ubiA PE=3 SV=2
121	C3K4C4	UBIA_PSEFS 4-hydroxybenzoate octaprenyltransferase OS=*Pseudomonas fluorescens* (strain SBW25) OX=216595 GN=ubiA PE=3 SV=1
122	Q7MZB6	UBIA_PHOLL 4-hydroxybenzoate octaprenyltransferase OS=*Photorhabdus laumondii* subsp. *laumondii* (strain DSM 15139 / CIP 105565 / TT01) OX=243265 GN=ubiA PE=3 SV=1
123	B2SF41	UBIA_FRATM 4-hydroxybenzoate octaprenyltransferase OS=*Francisella tularensis* subsp. *mediasiatica* (strain FSC147) OX=441952 GN=ubiA PE=3 SV=1
124	A6TGU9	UBIA_KLEP7 4-hydroxybenzoate octaprenyltransferase OS=*Klebsiella pneumoniae* subsp. *pneumoniae* (strain ATCC 700721 / MGH 78578) OX=272620 GN=ubiA PE=3 SV=1
125	A1RFH5	UBIA_SHESW 4-hydroxybenzoate octaprenyltransferase OS=*Shewanella* sp. (strain W3-18-1) OX=351745 GN=ubiA PE=3 SV=1
126	Q57GZ3	UBIA_SALCH 4-hydroxybenzoate octaprenyltransferase OS=*Salmonella choleraesuis* (strain SC-B67) OX=321314 GN=ubiA PE=3 SV=1
127	B4TDL7	UBIA_SALHS 4-hydroxybenzoate octaprenyltransferase OS=*Salmonella heidelberg* (strain SL476) OX=454169 GN=ubiA PE=3 SV=1
128	Q87F82	UBIA_XYLFT 4-hydroxybenzoate octaprenyltransferase OS=*Xylella fastidiosa* (strain Temecula1 / ATCC 700964) OX=183190 GN=ubiA PE=3 SV=1
129	A7MPP0	UBIA_CROS8 4-hydroxybenzoate octaprenyltransferase OS=*Cronobacter sakazakii* (strain ATCC BAA-894) OX=290339 GN=ubiA PE=3 SV=1
130	Q3K4G3	UBIA_PSEPF 4-hydroxybenzoate octaprenyltransferase OS=*Pseudomonas fluorescens* (strain Pf0-1) OX=205922 GN=ubiA PE=3 SV=1
131	A4W5F3	UBIA_ENT38 4-hydroxybenzoate octaprenyltransferase OS=*Enterobacter* sp. (strain 638) OX=399742 GN=ubiA PE=3 SV=1
132	Q5F9S8	UBIA_NEIG1 4-hydroxybenzoate octaprenyltransferase OS=*Neisseria gonorrhoeae* (strain ATCC 700825 / FA 1090) OX=242231 GN=ubiA PE=3 SV=1
133	A9N9X0	UBIA_COXBR 4-hydroxybenzoate octaprenyltransferase OS=*Coxiella burnetii* (strain RSA 331 / Henzerling II) OX=360115 GN=ubiA PE=3 SV=1
134	Q02E04	UBIA_PSEAB 4-hydroxybenzoate octaprenyltransferase OS=*Pseudomonas aeruginosa* (strain UCBPP-PA14) OX=208963 GN=ubiA PE=3 SV=1
135	A4VGN3	UBIA_PSEU5 4-hydroxybenzoate octaprenyltransferase OS=*Pseudomonas stutzeri* (strain A1501) OX=379731 GN=ubiA PE=3 SV=1
136	B2K1U4	UBIA_YERPB 4-hydroxybenzoate octaprenyltransferase OS=*Yersinia pseudotuberculosis* serotype IB (strain PB1/+) OX=502801 GN=ubiA PE=3 SV=1
137	A4YAV1	UBIA_SHEPC 4-hydroxybenzoate octaprenyltransferase OS=*Shewanella putrefaciens* (strain CN-32 / ATCC BAA-453) OX=319224 GN=ubiA PE=3 SV=1
138	Q1BYZ6	UBIA_BURCA 4-hydroxybenzoate octaprenyltransferase OS=*Burkholderia cenocepacia* (strain AU 1054) OX=331271 GN=ubiA PE=3 SV=1
139	A8GKB7	UBIA_SERP5 4-hydroxybenzoate octaprenyltransferase OS=*Serratia proteamaculans* (strain 568) OX=399741 GN=ubiA PE=3 SV=2
140	Q5ZVL0	UBIA_LEGPH 4-hydroxybenzoate octaprenyltransferase OS=*Legionella pneumophila* subsp. *pneumophila* (strain Philadelphia 1 / ATCC 33152 / DSM 7513) OX=272624 GN=ubiA PE=3 SV=1
141	B7UPK1	UBIA_ECO27 4-hydroxybenzoate octaprenyltransferase OS=*Escherichia coli* O127:H6 (strain E2348/69 / EPEC) OX=574521 GN=ubiA PE=3 SV=1
142	Q494A7	UBIA_BLOPB 4-hydroxybenzoate octaprenyltransferase OS=*Blochmannia pennsylvanicus* (strain BPEN) OX=291272 GN=ubiA PE=3 SV=1
143	C3LPT6	UBIA_VIBCM 4-hydroxybenzoate octaprenyltransferase OS=*Vibrio cholerae* serotype O1 (strain M66-2) OX=579112 GN=ubiA PE=3 SV=1
144	B5FQQ8	UBIA_SALDC 4-hydroxybenzoate octaprenyltransferase OS=*Salmonella dublin* (strain CT_02021853) OX=439851 GN=ubiA PE=3 SV=1
145	Q6LVS3	UBIA_PHOPR 4-hydroxybenzoate octaprenyltransferase OS=*Photobacterium profundum* (strain SS9) OX=298386 GN=ubiA PE=3 SV=1
146	Q1R3P6	UBIA_ECOUT 4-hydroxybenzoate octaprenyltransferase OS=*Escherichia coli* (strain UTI89 / UPEC) OX=364106 GN=ubiA PE=3 SV=1
147	A8FQF8	UBIA_SHESH 4-hydroxybenzoate octaprenyltransferase OS=*Shewanella sediminis* (strain HAW-EB3) OX=425104 GN=ubiA PE=3 SV=1
148	Q3BYM7	UBIA_XANC5 4-hydroxybenzoate octaprenyltransferase OS=*Xanthomonas campestris* pv. *vesicatoria* (strain 85-10) OX=316273 GN=ubiA PE=3 SV=1
149	Q39JF0	UBIA_BURL3 4-hydroxybenzoate octaprenyltransferase OS=*Burkholderia lata* (strain ATCC 17760 / DSM 23089 / LMG 22485 / NCIMB 9086 / R18194 / 383) OX=482957 GN=ubiA PE=3 SV=1
150	Q4K3L9	UBIA_PSEF5 4-hydroxybenzoate octaprenyltransferase OS=*Pseudomonas fluorescens* (strain ATCC BAA-477 / NRRL B-23932 / Pf-5) OX=220664 GN=ubiA PE=3 SV=1
151	B1JVZ5	UBIA_BURCC 4-hydroxybenzoate octaprenyltransferase OS=*Burkholderia cenocepacia* (strain MC0-3) OX=406425 GN=ubiA PE=3 SV=1
152	P57970	UBIA_PASMU 4-hydroxybenzoate octaprenyltransferase OS=*Pasteurella multocida* (strain Pm70) OX=272843 GN=ubiA PE=3 SV=1
153	Q0A5V9	UBIA_ALKEH 4-hydroxybenzoate octaprenyltransferase OS=*Alkalilimnicola ehrlichii* (strain ATCC BAA-1101 / DSM 17681 / MLHE-1) OX=187272 GN=ubiA PE=3 SV=1
154	A1AIL7	UBIA_ECOK1 4-hydroxybenzoate octaprenyltransferase OS=*Escherichia coli* O1:K1 / APEC OX=405955 GN=ubiA PE=3 SV=1
155	Q62H69	UBIA_BURMA 4-hydroxybenzoate octaprenyltransferase OS=*Burkholderia mallei* (strain ATCC 23344) OX=243160 GN=ubiA PE=3 SV=2
156	B6J612	UBIA_COXB1 4-hydroxybenzoate octaprenyltransferase OS=*Coxiella burnetii* (strain CbuK_Q154) OX=434924 GN=ubiA PE=3 SV=1
157	B1J454	UBIA_PSEPW 4-hydroxybenzoate octaprenyltransferase OS=*Pseudomonas putida* (strain W619) OX=390235 GN=ubiA PE=3 SV=1

158	Q3JBS8	UBIA_NITOC 4-hydroxybenzoate octaprenyltransferase OS=*Nitrosococcus oceani* (strain ATCC 19707 / BCRC 17464 / NCIMB 11848 / C-107) OX=323261 GN=ubiA PE=3 SV=1
159	Q12S01	UBIA_SHEDO 4-hydroxybenzoate octaprenyltransferase OS=*Shewanella denitrificans* (strain OS217 / ATCC BAA-1090 / DSM 15013) OX=318161 GN=ubiA PE=3 SV=2
160	Q7VQU0	UBIA_BLOFL 4-hydroxybenzoate octaprenyltransferase OS=*Blochmannia floridanus* OX=203907 GN=ubiA PE=3 SV=1
161	B5BJV7	UBIA_SALPK 4-hydroxybenzoate octaprenyltransferase OS=*Salmonella paratyphi* A (strain AKU_12601) OX=554290 GN=ubiA PE=3 SV=1
162	A5F4H4	UBIA_VIBC3 4-hydroxybenzoate octaprenyltransferase OS=*Vibrio cholerae* serotype O1 (strain ATCC 39541 / Classical Ogawa 395 / O395) OX=345073 GN=ubiA PE=3 SV=1
163	A9QYL1	UBIA_YERPG 4-hydroxybenzoate octaprenyltransferase OS=*Yersinia pestis* bv. Antiqua (strain Angola) OX=349746 GN=ubiA PE=3 SV=1
164	A7FN99	UBIA_YERP3 4-hydroxybenzoate octaprenyltransferase OS=*Yersinia pseudotuberculosis* serotype O:1b (strain IP 31758) OX=349747 GN=ubiA PE=3 SV=1
165	Q0BI45	UBIA_BURCM 4-hydroxybenzoate octaprenyltransferase OS=*Burkholderia ambifaria* (strain ATCC BAA-244 / AMMD) OX=339670 GN=ubiA PE=3 SV=1
166	A6WT76	UBIA_SHEB8 4-hydroxybenzoate octaprenyltransferase OS=*Shewanella baltica* (strain OS185) OX=402882 GN=ubiA PE=3 SV=1
167	A3QIF2	UBIA_SHELP 4-hydroxybenzoate octaprenyltransferase OS=*Shewanella loihica* (strain ATCC BAA-1088 / PV-4) OX=323850 GN=ubiA PE=3 SV=1
168	Q1CE95	UBIA_YERPN 4-hydroxybenzoate octaprenyltransferase OS=*Yersinia pestis* bv. Antiqua (strain Nepal516) OX=377628 GN=ubiA PE=3 SV=1
169	Q8EJJ5	UBIA_SHEON 4-hydroxybenzoate octaprenyltransferase OS=*Shewanella oneidensis* (strain MR-1) OX=211586 GN=ubiA PE=3 SV=1
170	A5IBS2	UBIA_LEGPC 4-hydroxybenzoate octaprenyltransferase OS=*Legionella pneumophila* (strain Corby) OX=400673 GN=ubiA PE=3 SV=1
171	A7ZUR2	UBIA_ECO24 4-hydroxybenzoate octaprenyltransferase OS=*Escherichia coli* O139:H28 (strain E24377A / ETEC) OX=331111 GN=ubiA PE=3 SV=1
172	Q1I2R3	UBIA_PSEE4 4-hydroxybenzoate octaprenyltransferase OS=*Pseudomonas entomophila* (strain L48) OX=384676 GN=ubiA PE=3 SV=1
173	P0AGK1	UBIA_ECOLI 4-hydroxybenzoate octaprenyltransferase OS=*Escherichia coli* (strain K12) OX=83333 GN=ubiA PE=1 SV=1
174	A8AN88	UBIA_CITK8 4-hydroxybenzoate octaprenyltransferase OS=*Citrobacter koseri* (strain ATCC BAA-895 / CDC 4225-83 / SGSC4696) OX=290338 GN=ubiA PE=3 SV=1
175	B0RMR4	UBIA_XANCB 4-hydroxybenzoate octaprenyltransferase OS=*Xanthomonas campestris* pv. campestris (strain B100) OX=509169 GN=ubiA PE=3 SV=1
176	B6I5Q5	UBIA_ECOSE 4-hydroxybenzoate octaprenyltransferase OS=*Escherichia coli* (strain SE11) OX=409438 GN=ubiA PE=3 SV=1
177	A1KSZ7	UBIA_NEIMF 4-hydroxybenzoate octaprenyltransferase OS=*Neisseria meningitidis* serogroup C / serotype 2a (strain ATCC 700532 / DSM 15464 / FAM18) OX=272831 GN=ubiA PE=3 SV=1
178	A1U6P5	UBIA_MARHV 4-hydroxybenzoate octaprenyltransferase OS=*Marinobacter hydrocarbonoclasticus* (strain ATCC 700491 / DSM 11845 / VT8) OX=351348 GN=ubiA PE=3 SV=1
179	B1IUL2	UBIA_ECOLC 4-hydroxybenzoate octaprenyltransferase OS=*Escherichia coli* (strain ATCC 8739 / DSM 1576 / Crooks) OX=481805 GN=ubiA PE=3 SV=1
180	Q8XGZ7	UBIA_SALTI 4-hydroxybenzoate octaprenyltransferase OS=*Salmonella typhi* OX=90370 GN=ubiA PE=3 SV=1
181	Q6D9I8	UBIA_PECAS 4-hydroxybenzoate octaprenyltransferase OS=*Pectobacterium atrosepticum* (strain SCRI 1043 / ATCC BAA-672) OX=218491 GN=ubiA PE=3 SV=1
182	Q1LJ64	UBIA_CUPMC 4-hydroxybenzoate octaprenyltransferase OS=*Cupriavidus metallidurans* (strain ATCC 43123 / DSM 2839 / NBRC 102507 / CH34) OX=266264 GN=ubiA PE=3 SV=1
183	A4Y0P0	UBIA_PSEMY 4-hydroxybenzoate octaprenyltransferase OS=*Pseudomonas mendocina* (strain ymp) OX=399739 GN=ubiA PE=3 SV=1
184	A1WVM9	UBIA_HALHL 4-hydroxybenzoate octaprenyltransferase OS=*Halorhodospira halophila* (strain DSM 244 / SL1) OX=349124 GN=ubiA PE=3 SV=2
185	Q9HTK0	UBIA_PSEAE 4-hydroxybenzoate octaprenyltransferase OS=*Pseudomonas aeruginosa* (strain ATCC 15692 / DSM 22644 / CIP 104116 / JCM 14847 / LMG 12228 / 1C / PRS 101 / PAO1) OX=208964 GN=ubiA PE=3 SV=1
186	Q4FR08	UBIA_PSYA2 4-hydroxybenzoate octaprenyltransferase OS=*Psychrobacter arcticus* (strain DSM 17307 / VKM B-2377 / 273-4) OX=259536 GN=ubiA PE=3 SV=1
187	B0BPA2	UBIA_ACTPJ 4-hydroxybenzoate octaprenyltransferase OS=*Actinobacillus pleuropneumoniae* serotype 3 (strain JL03) OX=434271 GN=ubiA PE=3 SV=1
188	B2I675	UBIA_XYLF2 4-hydroxybenzoate octaprenyltransferase OS=*Xylella fastidiosa* (strain M23) OX=405441 GN=ubiA PE=3 SV=1
189	B5QZ59	UBIA_SALEP 4-hydroxybenzoate octaprenyltransferase OS=*Salmonella enteritidis* PT4 (strain P125109) OX=550537 GN=ubiA PE=3 SV=1
190	B5F1Q3	UBIA_SALA4 4-hydroxybenzoate octaprenyltransferase OS=*Salmonella agona* (strain SL483) OX=454166 GN=ubiA PE=3 SV=1
191	Q5PL17	UBIA_SALPA 4-hydroxybenzoate octaprenyltransferase OS=*Salmonella paratyphi* A (strain ATCC 9150 / SARB42) OX=295319 GN=ubiA PE=3 SV=1
192	A1T087	UBIA_PSYIN 4-hydroxybenzoate octaprenyltransferase OS=*Psychromonas ingrahamii* (strain 37) OX=357804 GN=ubiA PE=3 SV=1
193	A6W3L5	UBIA_MARMS 4-hydroxybenzoate octaprenyltransferase OS=*Marinomonas* sp. (strain MWYL1) OX=400668 GN=ubiA PE=3 SV=1
194	B7V5P9	UBIA_PSEA8 4-hydroxybenzoate octaprenyltransferase OS=*Pseudomonas aeruginosa* (strain LESB58) OX=557722 GN=ubiA PE=3 SV=1
195	B2SS70	UBIA_XANOP 4-hydroxybenzoate octaprenyltransferase OS=*Xanthomonas oryzae* pv. oryzae (strain PXO99A) OX=360094 GN=ubiA PE=3 SV=1
196	Q0HQR8	UBIA_SHESR 4-hydroxybenzoate octaprenyltransferase OS=*Shewanella* sp. (strain MR-7) OX=60481 GN=ubiA PE=3 SV=1
197	Q83F93	UBIA_COXBU 4-hydroxybenzoate octaprenyltransferase OS=*Coxiella burnetii* (strain RSA 493 / Nine Mile phase I) OX=227377 GN=ubiA PE=3 SV=1

198	A4IYU3	UBIA_FRATW 4-hydroxybenzoate octaprenyltransferase OS=*Francisella tularensis* subsp. t*ularensis* (strain WY96-3418) OX=418136 GN=ubiA PE=3 SV=1
199	Q5WWR8	UBIA_LEGPL 4-hydroxybenzoate octaprenyltransferase OS=*Legionella pneumophila* (strain Lens) OX=297245 GN=ubiA PE=3 SV=1
200	Q5QUP3	UBIA_IDILO 4-hydroxybenzoate octaprenyltransferase OS=*Idiomarina loihiensis* (strain ATCC BAA-735 / DSM 15497 / L2-TR) OX=283942 GN=ubiA PE=3 SV=1
201	Q5H5R3	UBIA_XANOR 4-hydroxybenzoate octaprenyltransferase OS=*Xanthomonas oryzae* pv. oryzae (strain KACC10331 / KXO85) OX=291331 GN=ubiA PE=3 SV=1
202	A7NA50	UBIA_FRATF 4-hydroxybenzoate octaprenyltransferase OS=*Francisella tularensis* subsp. holarctica (strain FTNF002-00 / FTA) OX=458234 GN=ubiA PE=3 SV=1
203	C5B704	UBIA_EDWI9 4-hydroxybenzoate octaprenyltransferase OS=*Edwardsiella ictaluri* (strain 93-146) OX=634503 GN=ubiA PE=3 SV=1
204	A6VEG6	UBIA_PSEA7 4-hydroxybenzoate octaprenyltransferase OS=*Pseudomonas aeruginosa* (strain PA7) OX=381754 GN=ubiA PE=3 SV=1
205	O52366	UBIA_PROST 4-hydroxybenzoate octaprenyltransferase OS=*Providencia stuartii* OX=588 GN=ubiA PE=3 SV=1
206	Q7MQ87	UBIA_VIBVY 4-hydroxybenzoate octaprenyltransferase OS=*Vibrio vulnificus* (strain YJ016) OX=196600 GN=ubiA PE=3 SV=1
207	C1DK47	UBIA_AZOVD 4-hydroxybenzoate octaprenyltransferase OS=Azotobacter vinelandii (strain DJ / ATCC BAA-1303) OX=322710 GN=ubiA PE=3 SV=1
208	Q3YUU4	UBIA_SHISS 4-hydroxybenzoate octaprenyltransferase OS=*Shigella sonnei* (strain Ss046) OX=300269 GN=ubiA PE=3 SV=1
209	Q8PQD5	UBIA_XANAC 4-hydroxybenzoate octaprenyltransferase OS=*Xanthomonas axonopodis* pv. citri (strain 306) OX=190486 GN=ubiA PE=3 SV=1
210	B1XC39	UBIA_ECODH 4-hydroxybenzoate octaprenyltransferase OS=*Escherichia coli* (strain K12 / DH10B) OX=316385 GN=ubiA PE=3 SV=1
211	Q1GXB3	UBIA_METFK 4-hydroxybenzoate octaprenyltransferase OS=*Methylobacillus flagellatus* (strain KT / ATCC 51484 / DSM 6875) OX=265072 GN=ubiA PE=3 SV=1
212	Q31DM4	UBIA_HYDCU 4-hydroxybenzoate octaprenyltransferase OS=*Hydrogenovibrio crunogenus* (strain XCL-2) OX=317025 GN=ubiA PE=3 SV=1
213	A8GZR0	UBIA_SHEPA 4-hydroxybenzoate octaprenyltransferase OS=*Shewanella pealeana* (strain ATCC 700345 / ANG-SQ1) OX=398579 GN=ubiA PE=3 SV=1
214	Q7CPB4	UBIA_SALTY 4-hydroxybenzoate octaprenyltransferase OS=*Salmonella typhimurium* (strain LT2 / SGSC1412 / ATCC 700720) OX=99287 GN=ubiA PE=1 SV=1
215	B6ENU2	UBIA_ALISL 4-hydroxybenzoate octaprenyltransferase OS=*Aliivibrio salmonicida* (strain LFI1238) OX=316275 GN=ubiA PE=3 SV=1
216	B1LPK5	UBIA_ECOSM 4-hydroxybenzoate octaprenyltransferase OS=*Escherichia coli* (strain SMS-3-5 / SECEC) OX=439855 GN=ubiA PE=3 SV=1
217	A3CZR1	UBIA_SHEB5 4-hydroxybenzoate octaprenyltransferase OS=*Shewanella baltica* (strain OS155 / ATCC BAA-1091) OX=325240 GN=ubiA PE=3 SV=1
218	B1JNE6	UBIA_YERPY 4-hydroxybenzoate octaprenyltransferase OS=*Yersinia pseudotuberculosis* serotype O:3 (strain YPIII) OX=502800 GN=ubiA PE=3 SV=1
219	Q0SXQ7	UBIA_SHIF8 4-hydroxybenzoate octaprenyltransferase OS=*Shigella flexneri* serotype 5b (strain 8401) OX=373384 GN=ubiA PE=3 SV=1
220	B7VMA0	UBIA_VIBA3 4-hydroxybenzoate octaprenyltransferase OS=*Vibrio atlanticus* (strain LGP32) OX=575788 GN=ubiA PE=3 SV=1
221	Q1QSG2	UBIA_CHRSD 4-hydroxybenzoate octaprenyltransferase OS=*Chromohalobacter salexigens* (strain ATCC BAA-138 / DSM 3043 / CIP 106854 / NCIMB 13768 / 1H11) OX=290398 GN=ubiA PE=3 SV=1
222	Q87U39	UBIA_PSESM 4-hydroxybenzoate octaprenyltransferase OS=*Pseudomonas syringae* pv. tomato (strain ATCC BAA-871 / DC3000) OX=223283 GN=ubiA PE=3 SV=1
223	A5WB34	UBIA_PSEP1 4-hydroxybenzoate octaprenyltransferase OS=*Pseudomonas putida* (strain ATCC 700007 / DSM 6899 / BCRC 17059 / F1) OX=351746 GN=ubiA PE=3 SV=1
224	Q15ZT9	UBIA_PSEA6 4-hydroxybenzoate octaprenyltransferase OS=*Pseudoalteromonas atlantica* (strain T6c / ATCC BAA-1087) OX=342610 GN=ubiA PE=3 SV=2
225	A9MH90	UBIA_SALAR 4-hydroxybenzoate octaprenyltransferase OS=*Salmonella arizonae* (strain ATCC BAA-731 / CDC346-86 / RSK2980) OX=41514 GN=ubiA PE=3 SV=1
226	Q48AL9	UBIA_COLP3 4-hydroxybenzoate octaprenyltransferase OS=*Colwellia psychrerythraea* (strain 34H / ATCC BAA-681) OX=167879 GN=ubiA PE=3 SV=1
227	A9M390	UBIA_NEIM0 4-hydroxybenzoate octaprenyltransferase OS=*Neisseria meningitidis* serogroup C (strain 053442) OX=374833 GN=ubiA PE=3 SV=1
228	Q9JV93	UBIA_NEIMA 4-hydroxybenzoate octaprenyltransferase OS=*Neisseria meningitidis* serogroup A / serotype 4A (strain DSM 15465 / Z2491) OX=122587 GN=ubiA PE=3 SV=1
229	A0KEP9	UBIA_AERHH 4-hydroxybenzoate octaprenyltransferase OS=*Aeromonas hydrophila* subsp. hydrophila (strain ATCC 7966 / DSM 30187 / JCM 1027 / KCTC 2358 / NCIMB 9240) OX=380703 GN=ubiA PE=3 SV=1
230	Q5NGI4	UBIA_FRATT 4-hydroxybenzoate octaprenyltransferase OS=*Francisella tularensis* subsp. tularensis (strain SCHU S4 / Schu 4) OX=177416 GN=ubiA PE=3 SV=1
231	Q7CKP5	UBIA_YERPE 4-hydroxybenzoate octaprenyltransferase OS=*Yersinia pestis* OX=632 GN=ubiA PE=3 SV=1
232	C0Q4D9	UBIA_SALPC 4-hydroxybenzoate octaprenyltransferase OS=Salmonella paratyphi C (strain RKS4594) OX=476213 GN=ubiA PE=3 SV=1
233	B7MJ30	UBIA_ECO45 4-hydroxybenzoate octaprenyltransferase OS=*Escherichia coli* O45:K1 (strain S88 / ExPEC) OX=585035 GN=ubiA PE=3 SV=1
234	Q3JNX1	UBIA_BURP1 4-hydroxybenzoate octaprenyltransferase OS=*Burkholderia pseudomallei* (strain 1710b) OX=320372 GN=ubiA PE=3 SV=2
235	Q7VL38	UBIA_HAEDU 4-hydroxybenzoate octaprenyltransferase OS=*Haemophilus ducreyi* (strain 35000HP / ATCC 700724) OX=233412 GN=ubiA PE=3 SV=1
236	B7NS04	UBIA_ECO7I 4-hydroxybenzoate octaprenyltransferase OS=*Escherichia coli* O7:K1 (strain IAI39 / ExPEC) OX=585057 GN=ubiA PE=3 SV=1
237	Q8DD49	UBIA_VIBVU 4-hydroxybenzoate octaprenyltransferase OS=*Vibrio vulnificus* (strain CMCP6) OX=216895 GN=ubiA PE=3 SV=1

238	Q2NR07	UBIA_SODGM 4-hydroxybenzoate octaprenyltransferase OS=*Sodalis glossinidius* (strain morsitans) OX=343509 GN=ubiA PE=3 SV=1
239	B7M7V3	UBIA_ECO8A 4-hydroxybenzoate octaprenyltransferase OS=*Escherichia coli* O8 (strain IAI1) OX=585034 GN=ubiA PE=3 SV=1
240	A0Q4X3	UBIA_FRATN 4-hydroxybenzoate octaprenyltransferase OS=*Francisella tularensis* subsp. novicida (strain U112) OX=401614 GN=ubiA PE=3 SV=1
241	Q12GA4	UBIA_POLSJ 4-hydroxybenzoate octaprenyltransferase OS=*Polaromonas* sp. (strain JS666 / ATCC BAA-500) OX=296591 GN=ubiA PE=3 SV=1
242	Q21DY7	UBIA_SACD2 4-hydroxybenzoate octaprenyltransferase OS=*Saccharophagus degradans* (strain 2-40 / ATCC 43961 / DSM 17024) OX=203122 GN=ubiA PE=3 SV=1
243	Q83IP7	UBIA_SHIFL 4-hydroxybenzoate octaprenyltransferase OS=*Shigella flexneri* OX=623 GN=ubiA PE=3 SV=1
244	Q9KVP7	UBIA_VIBCH 4-hydroxybenzoate octaprenyltransferase OS=*Vibrio cholerae* serotype O1 (strain ATCC 39315 / El Tor Inaba N16961) OX=243277 GN=ubiA PE=3 SV=1
245	A4JBP9	UBIA_BURVG 4-hydroxybenzoate octaprenyltransferase OS=*Burkholderia vietnamiensis* (strain G4 / LMG 22486) OX=269482 GN=ubiA PE=3 SV=1
246	Q5X5D5	UBIA_LEGPA 4-hydroxybenzoate octaprenyltransferase OS=*Legionella pneumophila* (strain Paris) OX=297246 GN=ubiA PE=3 SV=1
247	B7LAY8	UBIA_ECO55 4-hydroxybenzoate octaprenyltransferase OS=*Escherichia coli* (strain 55989 / EAEC) OX=585055 GN=ubiA PE=3 SV=1
248	Q2SNV5	UBIA_HAHCH 4-hydroxybenzoate octaprenyltransferase OS=*Hahella chejuensis* (strain KCTC 2396) OX=349521 GN=ubiA PE=3 SV=1
249	B0KQB7	UBIA_PSEPG 4-hydroxybenzoate octaprenyltransferase OS=*Pseudomonas putida* (strain GB-1) OX=76869 GN=ubiA PE=3 SV=1
250	B3H1F9	UBIA_ACTP7 4-hydroxybenzoate octaprenyltransferase OS=*Actinobacillus pleuropneumoniae* serotype 7 (strain AP76) OX=537457 GN=ubiA PE=3 SV=1
251	B1YTH7	UBIA_BURA4 4-hydroxybenzoate octaprenyltransferase OS=*Burkholderia ambifaria* (strain MC40-6) OX=398577 GN=ubiA PE=3 SV=1
252	A4TRU8	UBIA_YERPP 4-hydroxybenzoate octaprenyltransferase OS=*Yersinia pestis* (strain Pestoides F) OX=386656 GN=ubiA PE=3 SV=1
253	Q0TA22	UBIA_ECOL5 4-hydroxybenzoate octaprenyltransferase OS=*Escherichia coli* O6:K15:H31 (strain 536 / UPEC) OX=362663 GN=ubiA PE=3 SV=1
254	A9N1L3	UBIA_SALPB 4-hydroxybenzoate octaprenyltransferase OS=Salmonella paratyphi B (strain ATCC BAA-1250 / SPB7) OX=1016998 GN=ubiA PE=3 SV=1
255	A9AGE2	UBIA_BURM1 4-hydroxybenzoate octaprenyltransferase OS=Burkholderia multivorans (strain ATCC 17616 / 249) OX=395019 GN=ubiA PE=3 SV=1
256	Q4UZN6	UBIA_XANC8 4-hydroxybenzoate octaprenyltransferase OS=Xanthomonas campestris pv. campestris (strain 8004) OX=314565 GN=ubiA PE=3 SV=1
257	Q14HY6	UBIA_FRAT1 4-hydroxybenzoate octaprenyltransferase OS=*Francisella tularensis* subsp. *tularensis* (strain FSC 198) OX=393115 GN=ubiA PE=3 SV=1
258	Q145H3	UBIA_PARXL 4-hydroxybenzoate octaprenyltransferase OS=*Paraburkholderia xenovorans* (strain LB400) OX=266265 GN=ubiA PE=3 SV=1
259	A1TJR2	UBIA_ACIAC 4-hydroxybenzoate octaprenyltransferase OS=*Acidovorax citrulli* (strain AAC00-1) OX=397945 GN=ubiA PE=3 SV=1
260	B4TQQ2	UBIA_SALSV 4-hydroxybenzoate octaprenyltransferase OS=*Salmonella schwarzengrund* (strain CVM19633) OX=439843 GN=ubiA PE=3 SV=1
261	B0U042	UBIA_FRAP2 4-hydroxybenzoate octaprenyltransferase OS=*Francisella philomiragia* subsp. philomiragia (strain ATCC 25017 / FSC 153 / O#319-036) OX=484022 GN=ubiA PE=3 SV=1
262	A1WKC0	UBIA_VEREI 4-hydroxybenzoate octaprenyltransferase OS=*Verminephrobacter eiseniae* (strain EF01-2) OX=391735 GN=ubiA PE=3 SV=1
263	A7N0X0	UBIA_VIBCB 4-hydroxybenzoate octaprenyltransferase OS=*Vibrio campbellii* (strain ATCC BAA-1116 / BB120) OX=338187 GN=ubiA PE=3 SV=1
264	B5R7T3	UBIA_SALG2 4-hydroxybenzoate octaprenyltransferase OS=*Salmonella gallinarum* (strain 287/91 / NCTC 13346) OX=550538 GN=ubiA PE=3 SV=1
265	Q46XG9	UBIA_CUPPJ 4-hydroxybenzoate octaprenyltransferase OS=*Cupriavidus pinatubonensis* (strain JMP 134 / LMG 1197) OX=264198 GN=ubiA PE=3 SV=1
266	Q8PDE8	UBIA_XANCP 4-hydroxybenzoate octaprenyltransferase OS=*Xanthomonas campestris* pv. campestris (strain ATCC 33913 / DSM 3586 / NCPPB 528 / LMG 568 / P 25) OX=190485 GN=ubiA PE=3 SV=1
267	Q1Q996	UBIA_PSYCK 4-hydroxybenzoate octaprenyltransferase OS=*Psychrobacter cryohalolentis* (strain ATCC BAA-1226 / DSM 17306 / VKM B-2378 / K5) OX=335284 GN=ubiA PE=3 SV=1
268	B9MBT5	UBIA_ACIET 4-hydroxybenzoate octaprenyltransferase OS=*Acidovorax ebreus* (strain TPSY) OX=535289 GN=ubiA PE=3 SV=1
269	A1W307	UBIA_ACISJ 4-hydroxybenzoate octaprenyltransferase OS=*Acidovorax* sp. (strain JS42) OX=232721 GN=ubiA PE=3 SV=1
270	A3N0I2	UBIA_ACTP2 4-hydroxybenzoate octaprenyltransferase OS=Actinobacillus pleuropneumoniae serotype 5b (strain L20) OX=416269 GN=ubiA PE=3 SV=1
271	C4L7X6	UBIA_TOLAT 4-hydroxybenzoate octaprenyltransferase OS=*Tolumonas auensis (*strain DSM 9187 / TA4) OX=595494 GN=ubiA PE=3 SV=1
272	B8E613	UBIA_SHEB2 4-hydroxybenzoate octaprenyltransferase OS=*Shewanella baltica* (strain OS223) OX=407976 GN=ubiA PE=3 SV=1
273	Q9K083	UBIA_NEIMB 4-hydroxybenzoate octaprenyltransferase OS=*Neisseria meningitidis* serogroup B (strain MC58) OX=122586 GN=ubiA PE=3 SV=1
274	B2TX76	UBIA_SHIB3 4-hydroxybenzoate octaprenyltransferase OS=*Shigella boydii* serotype 18 (strain CDC 3083-94 / BS512) OX=344609 GN=ubiA PE=3 SV=1
275	A9L4Q8	UBIA_SHEB9 4-hydroxybenzoate octaprenyltransferase OS=*Shewanella baltica* (strain OS195) OX=399599 GN=ubiA PE=3 SV=1
276	A4G1Y9	UBIA_HERAR 4-hydroxybenzoate octaprenyltransferase OS=Herminiimonas arsenicoxydans OX=204773 GN=ubiA PE=3 SV=1
277	B1XRV6	UBIA_POLNS 4-hydroxybenzoate octaprenyltransferase OS=Polynucleobacter necessarius subsp. necessarius (strain STIR1) OX=452638 GN=ubiA PE=3 SV=1

278	A9KGW7	UBIA_COXBN 4-hydroxybenzoate octaprenyltransferase OS=Coxiella burnetii (strain Dugway 5J108-111) OX=434922 GN=ubiA PE=3 SV=1
279	Q0VTA7	UBIA_ALCBS 4-hydroxybenzoate octaprenyltransferase OS=*Alcanivorax borkumensis* (strain ATCC 700651 / DSM 11573 / NCIMB 13689 / SK2) OX=393595 GN=ubiA PE=3 SV=1
280	A1S2M4	UBIA_SHEAM 4-hydroxybenzoate octaprenyltransferase OS=*Shewanella amazonensis* (strain ATCC BAA-1098 / SB2B) OX=326297 GN=ubiA PE=3 SV=1
281	A2SMA7	UBIA_METPP 4-hydroxybenzoate octaprenyltransferase OS=*Methylibium petroleiphilum* (strain ATCC BAA-1232 / LMG 22953 / PM1) OX=420662 GN=ubiA PE=3 SV=1
282	Q21S73	UBIA_RHOFT 4-hydroxybenzoate octaprenyltransferase OS=*Rhodoferax ferrireducens* (strain ATCC BAA-621 / DSM 15236 / T118) OX=338969 GN=ubiA PE=3 SV=1
283	Q2Y5S3	UBIA_NITMU 4-hydroxybenzoate octaprenyltransferase OS=*Nitrosospira multiformis* (strain ATCC 25196 / NCIMB 11849 / C 71) OX=323848 GN=ubiA PE=3 SV=1
284	Q4ZLB4	UBIA_PSEU2 4-hydroxybenzoate octaprenyltransferase OS=*Pseudomonas syringae* pv. syringae (strain B728a) OX=205918 GN=ubiA PE=3 SV=1
285	Q327U7	UBIA_SHIDS 4-hydroxybenzoate octaprenyltransferase OS=*Shigella dysenteriae* serotype 1 (strain Sd197) OX=300267 GN=ubiA PE=3 SV=1
286	B0U2A2	UBIA_XYLFM 4-hydroxybenzoate octaprenyltransferase OS=*Xylella fastidiosa* (strain M12) OX=405440 GN=ubiA PE=3 SV=1
287	Q5E207	UBIA_ALIF1 4-hydroxybenzoate octaprenyltransferase OS=*Aliivibrio fischeri* (strain ATCC 700601 / ES114) OX=312309 GN=ubiA PE=3 SV=1
288	Q87KM9	UBIA_VIBPA 4-hydroxybenzoate octaprenyltransferase OS=*Vibrio parahaemolyticus* serotype O3:K6 (strain RIMD 2210633) OX=223926 GN=ubiA PE=3 SV=1
289	P0AGK2	UBIA_ECOL6 4-hydroxybenzoate octaprenyltransferase OS=*Escherichia coli* O6:H1 (strain CFT073 / ATCC 700928 / UPEC) OX=199310 GN=ubiA PE=3 SV=1
290	B4RK00	UBIA_NEIG2 4-hydroxybenzoate octaprenyltransferase OS=*Neisseria gonorrhoeae* (strain NCCP11945) OX=521006 GN=ubiA PE=3 SV=1
291	Q6F9N8	UBIA_ACIAD 4-hydroxybenzoate octaprenyltransferase OS=*Acinetobacter bayly*i (strain ATCC 33305 / BD413 / ADP1) OX=62977 GN=ubiA PE=3 SV=2
292	Q31TU8	UBIA_SHIBS 4-hydroxybenzoate octaprenyltransferase OS=*Shigella boydii* serotype 4 (strain Sb227) OX=300268 GN=ubiA PE=3 SV=1
293	A0KSE2	UBIA_SHESA 4-hydroxybenzoate octaprenyltransferase OS=*Shewanella* sp. (strain ANA-3) OX=94122 GN=ubiA PE=3 SV=1
294	C6DKC9	UBIA_PECCP 4-hydroxybenzoate octaprenyltransferase OS=*Pectobacterium carotovorum* subsp. carotovorum (strain PC1) OX=561230 GN=ubiA PE=3 SV=1
295	Q0I0W3	UBIA_HAES1 4-hydroxybenzoate octaprenyltransferase OS=*Haemophilus somnus* (strain 129Pt) OX=205914 GN=ubiA PE=3 SV=1
296	Q5P5M0	UBIA_AROAE 4-hydroxybenzoate octaprenyltransferase OS=*Aromatoleum aromaticum* (strain EbN1) OX=76114 GN=ubiA PE=3 SV=2
297	A4STB5	UBIA_AERS4 4-hydroxybenzoate octaprenyltransferase OS=*Aeromonas salmonicida* (strain A449) OX=382245 GN=ubiA PE=3 SV=2
298	Q0BNI0	UBIA_FRATO 4-hydroxybenzoate octaprenyltransferase OS=*Francisella tularensis* subsp. holarctica (strain OSU18) OX=393011 GN=ubiA PE=3 SV=1
299	A5CXV6	UBIA_VESOH 4-hydroxybenzoate octaprenyltransferase OS=*Vesicomyosocius okutanii* subsp. Calyptogena okutanii (strain HA) OX=412965 GN=ubiA PE=3 SV=2
300	P0AGK3	UBIA_ECO57 4-hydroxybenzoate octaprenyltransferase OS=*Escherichia coli* O157:H7 OX=83334 GN=ubiA PE=3 SV=1
301	A0K4Q5	UBIA_BURCH 4-hydroxybenzoate octaprenyltransferase OS=*Burkholderia cenocepacia* (strain HI2424) OX=331272 GN=ubiA PE=3 SV=1
302	Q63R13	UBIA_BURPS 4-hydroxybenzoate octaprenyltransferase OS=*Burkholderia pseudomallei* (strain K96243) OX=272560 GN=ubiA PE=3 SV=2
303	B4T1S9	UBIA_SALNS 4-hydroxybenzoate octaprenyltransferase OS=*Salmonella newport* (strain SL254) OX=423368 GN=ubiA PE=3 SV=1
304	B0UUY3	UBIA_HISS2 4-hydroxybenzoate octaprenyltransferase OS=*Histophilus somni* (strain 2336) OX=228400 GN=ubiA PE=3 SV=1
305	Q2A564	UBIA_FRATH 4-hydroxybenzoate octaprenyltransferase OS=*Francisella tularensis* subsp. holarctica (strain LVS) OX=376619 GN=ubiA PE=3 SV=1
306	Q0AGZ9	UBIA_NITEC 4-hydroxybenzoate octaprenyltransferase OS=*Nitrosomonas eutropha* (strain DSM 101675 / C91 / Nm57) OX=335283 GN=ubiA PE=3 SV=1
307	Q608Z9	UBIA_METCA 4-hydroxybenzoate octaprenyltransferase OS=*Methylococcus capsulatus* (strain ATCC 33009 / NCIMB 11132 / Bath) OX=243233 GN=ubiA PE=3 SV=1
308	A1JRU3	UBIA_YERE8 4-hydroxybenzoate octaprenyltransferase OS=*Yersinia enterocolitica* serotype O:8 / biotype 1B (strain NCTC 13174 / 8081) OX=393305 GN=ubiA PE=3 SV=1
309	B7LL12	UBIA_ESCF3 4-hydroxybenzoate octaprenyltransferase OS=*Escherichia fergusonii* (strain ATCC 35469 / DSM 13698 / CDC 0568-73) OX=585054 GN=ubiA PE=3 SV=1
310	A1VJ21	UBIA_POLNA 4-hydroxybenzoate octaprenyltransferase OS=*Polaromonas naphthalenivorans* (strain CJ2) OX=365044 GN=ubiA PE=3 SV=1
311	Q2P8F5	UBIA_XANOM 4-hydroxybenzoate octaprenyltransferase OS=*Xanthomonas oryzae* pv. oryzae (strain MAFF 311018) OX=342109 GN=ubiA PE=3 SV=1
312	Q07XN2	UBIA_SHEFN 4-hydroxybenzoate octaprenyltransferase OS=*Shewanella frigidimarina* (strain NCIMB 400) OX=318167 GN=ubiA PE=3 SV=1
313	Q3SLJ4	UBIA_THIDA 4-hydroxybenzoate octaprenyltransferase OS=*Thiobacillus denitrificans* (strain ATCC 25259) OX=292415 GN=ubiA PE=3 SV=1
314	B7MRH2	UBIA_ECO81 4-hydroxybenzoate octaprenyltransferase OS=*Escherichia coli* O81 (strain ED1a) OX=585397 GN=ubiA PE=3 SV=1
315	A6SUU0	UBIA_JANMA 4-hydroxybenzoate octaprenyltransferase OS=*Janthinobacterium* sp. (strain Marseille) OX=375286 GN=ubiA PE=3 SV=1
316	Q0HN13	UBIA_SHESM 4-hydroxybenzoate octaprenyltransferase OS=*Shewanella* sp. (strain MR-4) OX=60480 GN=ubiA PE=3 SV=1
317	A3MPC2	UBIA_BURM7 4-hydroxybenzoate octaprenyltransferase OS=*Burkholderia mallei* (strain NCTC 10247) OX=320389 GN=ubiA PE=3 SV=1

318	Q0K739	UBIA_CUPNH 4-hydroxybenzoate octaprenyltransferase OS=*Cupriavidus necator* (strain ATCC 17699 / H16 / DSM 428 / Stanier 337) OX=381666 GN=ubiA PE=3 SV=2
319	B6J375	UBIA_COXB2 4-hydroxybenzoate octaprenyltransferase OS=*Coxiella burnetii* (strain CbuG_Q212) OX=434923 GN=ubiA PE=3 SV=1
320	A1K2P1	UBIA_AZOSB 4-hydroxybenzoate octaprenyltransferase OS=*Azoarcus* sp. (strain BH72) OX=418699 GN=ubiA PE=3 SV=1
321	B5FCB4	UBIA_ALIFM 4-hydroxybenzoate octaprenyltransferase OS=*Aliivibrio fischeri* (strain MJ11) OX=388396 GN=ubiA PE=3 SV=1
322	A3NZ16	UBIA_BURP0 4-hydroxybenzoate octaprenyltransferase OS=*Burkholderia pseudomallei* (strain 1106a) OX=357348 GN=ubiA PE=3 SV=1
323	B7NFY4	UBIA_ECOLU 4-hydroxybenzoate octaprenyltransferase OS=*Escherichia coli* O17:K52:H18 (strain UMN026 / ExPEC) OX=585056 GN=ubiA PE=3 SV=1
324	Q8XVY9	UBIA_RALSO 4-hydroxybenzoate octaprenyltransferase OS=Ralstonia solanacearum (strain GMI1000) OX=267608 GN=ubiA PE=3 SV=1
325	Q88C65	UBIA_PSEPK 4-hydroxybenzoate octaprenyltransferase OS=*Pseudomonas putida* (strain ATCC 47054 / DSM 6125 / NCIMB 11950 / KT2440) OX=160488 GN=ubiA PE=3 SV=1
326	B5Z181	UBIA_ECO5E 4-hydroxybenzoate octaprenyltransferase OS=*Escherichia coli* O157:H7 (strain EC4115 / EHEC) OX=444450 GN=ubiA PE=3 SV=1
327	B2JGG3	UBIA_PARP8 4-hydroxybenzoate octaprenyltransferase OS=*Paraburkholderia phymatum* (strain DSM 17167 / CIP 108236 / LMG 21445 / STM815) OX=391038 GN=ubiA PE=3 SV=1
328	A3NDA6	UBIA_BURP6 4-hydroxybenzoate octaprenyltransferase OS=*Burkholderia pseudomallei* (strain 668) OX=320373 GN=ubiA PE=3 SV=1
329	Q48BQ5	UBIA_PSE14 4-hydroxybenzoate octaprenyltransferase OS=*Pseudomonas savastanoi* pv. phaseolicola (strain 1448A / Race 6) OX=264730 GN=ubiA PE=3 SV=1
330	A4SZT5	UBIA_POLAQ 4-hydroxybenzoate octaprenyltransferase OS=*Polynucleobacter asymbioticus* (strain DSM 18221 / CIP 109841 / QLW-P1DMWA-1) OX=312153 GN=ubiA PE=3 SV=1
331	B8CTT5	UBIA_SHEPW 4-hydroxybenzoate octaprenyltransferase OS=*Shewanella piezotolerans* (strain WP3 / JCM 13877) OX=225849 GN=ubiA PE=3 SV=1
332	Q2SZ16	UBIA_BURTA 4-hydroxybenzoate octaprenyltransferase OS=*Burkholderia thailandensis* (strain ATCC 700388 / DSM 13276 / CIP 106301 / E264) OX=271848 GN=ubiA PE=3 SV=2
333	Q3IHW5	UBIA_PSET1 4-hydroxybenzoate octaprenyltransferase OS=*Pseudoalteromonas translucida* (strain TAC 125) OX=326442 GN=ubiA PE=3 SV=1
334	C5A133	UBIA_ECOBW 4-hydroxybenzoate octaprenyltransferase OS=*Escherichia coli* (strain K12 / MC4100 / BW2952) OX=595496 GN=ubiA PE=3 SV=1
335	B5XXZ2	UBIA_KLEP3 4-hydroxybenzoate octaprenyltransferase OS=*Klebsiella pneumoniae* (strain 342) OX=507522 GN=ubiA PE=3 SV=1
336	Q82TP2	UBIA_NITEU 4-hydroxybenzoate octaprenyltransferase OS=*Nitrosomonas europaea* (strain ATCC 19718 / CIP 103999 / KCTC 2705 / NBRC 14298) OX=228410 GN=ubiA PE=3 SV=2
337	A8A7E0	UBIA_ECOHS 4-hydroxybenzoate octaprenyltransferase OS=*Escherichia coli* O9:H4 (strain HS) OX=331112 GN=ubiA PE=3 SV=1
338	Q47J11	UBIA_DECAR 4-hydroxybenzoate octaprenyltransferase OS=*Dechloromonas aromatica* (strain RCB) OX=159087 GN=ubiA PE=3 SV=1
339	A8F1B6	COXX_RICM5 Protoheme IX farnesyltransferase OS=*Rickettsia massiliae* (strain Mtu5) OX=416276 GN=ctaB PE=3 SV=2
340	A6U6U6	COXX_SINMW Protoheme IX farnesyltransferase OS=*Sinorhizobium medicae* (strain WSM419) OX=366394 GN=ctaB PE=3 SV=1
341	Q2IPE5	COXX_ANADE Protoheme IX farnesyltransferase OS=Anaeromyxobacter dehalogenans (strain 2CP-C) OX=290397 GN=ctaB PE=3 SV=2
342	Q1BTD0	COXX_BURCA Protoheme IX farnesyltransferase OS=*Burkholderia cenocepacia* (strain AU 1054) OX=331271 GN=ctaB PE=3 SV=1
343	Q6G4C6	COXX_BARHE Protoheme IX farnesyltransferase OS=*Bartonella henselae* (strain ATCC 49882 / DSM 28221 / Houston 1) OX=283166 GN=ctaB PE=3 SV=1
344	Q325H3	CYOE_SHIBS Protoheme IX farnesyltransferase OS=*Shigella boydii* serotype 4 (strain Sb227) OX=300268 GN=cyoE PE=3 SV=1
345	A5CXZ0	CYOE_VESOH Protoheme IX farnesyltransferase OS=Vesicomyosocius okutanii subsp. Calyptogena okutanii (strain HA) OX=412965 GN=cyoE PE=3 SV=1
346	Q3K773	CYOE2_PSEPF Protoheme IX farnesyltransferase 2 OS=*Pseudomonas fluorescens* (strain Pf0-1) OX=205922 GN=cyoE2 PE=3 SV=1
347	P0AEA5	CYOE_ECOLI Protoheme IX farnesyltransferase OS=*Escherichia coli* (strain K12) OX=83333 GN=cyoE PE=1 SV=1
348	Q12IC7	CYOE_SHEDO Protoheme IX farnesyltransferase OS=*Shewanella denitrificans* (strain OS217 / ATCC BAA-1090 / DSM 15013) OX=318161 GN=cyoE PE=3 SV=1
349	B1JDU9	CYOE2_PSEPW Protoheme IX farnesyltransferase 2 OS=*Pseudomonas putida* (strain W619) OX=390235 GN=cyoE2 PE=3 SV=1
350	A7FLD4	CYOE_YERP3 Protoheme IX farnesyltransferase OS=*Yersinia pseudotuberculosis* serotype O:1b (strain IP 31758) OX=349747 GN=cyoE PE=3 SV=1
351	Q87IH5	CYOE2_VIBPA Protoheme IX farnesyltransferase 2 OS=*Vibrio parahaemolyticus* serotype O3:K6 (strain RIMD 2210633) OX=223926 GN=cyoE2 PE=3 SV=1
352	Q3J6R6	CYOE_NITOC Protoheme IX farnesyltransferase OS=*Nitrosococcus oceani* (strain ATCC 19707 / BCRC 17464 / NCIMB 11848 / C-107) OX=323261 GN=cyoE PE=3 SV=1
353	P44739	MENA_HAEIN 1,4-dihydroxy-2-naphthoate octaprenyltransferase OS=*Haemophilus influenzae* (strain ATCC 51907 / DSM 11121 / KW20 / Rd) OX=71421 GN=menA PE=3 SV=1
354	P32166	MENA_ECOLI 1,4-dihydroxy-2-naphthoate octaprenyltransferase OS=*Escherichia coli* (strain K12) OX=83333 GN=menA PE=1 SV=1
355	Q1H1S8	COXX_METFK Protoheme IX farnesyltransferase OS=*Methylobacillus flagellatus* (strain KT / ATCC 51484 / DSM 6875) OX=265072 GN=ctaB PE=3 SV=1
356	Q2IR91	COXX_RHOP2 Protoheme IX farnesyltransferase OS=*Rhodopseudomonas palustris* (strain HaA2) OX=316058 GN=ctaB PE=3 SV=1
357	Q3SLW5	COXX_THIDA Protoheme IX farnesyltransferase OS=*Thiobacillus denitrificans* (strain ATCC 25259) OX=292415 GN=ctaB PE=3 SV=1

358	P9WIP2	MENA_MYCTO 1,4-dihydroxy-2-naphthoate octaprenyltransferase OS=*Mycobacterium tuberculosis* (strain CDC 1551 / Oshkosh) OX=83331 GN=menA PE=3 SV=1
359	P9WIP3	MENA_MYCTU 1,4-dihydroxy-2-naphthoate octaprenyltransferase OS=*Mycobacterium tuberculosis* (strain ATCC 25618 / H37Rv) OX=83332 GN=menA PE=1 SV=1
360	Q7U021	COXX_MYCBO Protoheme IX farnesyltransferase OS=*Mycobacterium bovis* (strain ATCC BAA-935 / AF2122/97) OX=233413 GN=ctaB PE=3 SV=1
361	Q1B9B1	COXX_MYCSS Protoheme IX farnesyltransferase OS=*Mycobacterium* sp. (strain MCS) OX=164756 GN=ctaB PE=3 SV=2
362	Q83NK0	COXX_TROW8 Protoheme IX farnesyltransferase OS=*Tropheryma whipplei* (strain TW08/27) OX=218496 GN=ctaB PE=3 SV=1
363	P65651	MENA_MYCBO 1,4-dihydroxy-2-naphthoate octaprenyltransferase OS=*Mycobacterium bovis* (strain ATCC BAA-935 / AF2122/97) OX=233413 GN=menA PE=3 SV=1
364	O07134	MENA_MYCLE 1,4-dihydroxy-2-naphthoate octaprenyltransferase OS=*Mycobacterium leprae* (strain TN) OX=272631 GN=menA PE=3 SV=1
365	A3PZB2	COXX_MYCSJ Protoheme IX farnesyltransferase OS=*Mycobacterium* sp. (strain JLS) OX=164757 GN=ctaB PE=3 SV=1
366	Q4JVK2	COXX_CORJK Protoheme IX farnesyltransferase OS=*Corynebacterium jeikeium* (strain K411) OX=306537 GN=ctaB PE=3 SV=1
367	A1UFQ0	COXX_MYCSK Protoheme IX farnesyltransferase OS=*Mycobacterium* sp. (strain KMS) OX=189918 GN=ctaB PE=3 SV=2
368	A5CRS6	COXX_CLAM3 Protoheme IX farnesyltransferase OS=*Clavibacter michiganensis* subsp. michiganensis (strain NCPPB 382) OX=443906 GN=ctaB PE=3 SV=1
369	A4FBP2	COXX2_SACEN Protoheme IX farnesyltransferase 2 OS=*Saccharopolyspora erythraea* (strain ATCC 11635 / DSM 40517 / JCM 4748 / NBRC 13426 / NCIMB 8594 / NRRL 2338) OX=405948 GN=ctaB2 PE=3 SV=1
370	B8H7N5	COXX_PSECP Protoheme IX farnesyltransferase OS=*Pseudarthrobacter chlorophenolicus* (strain ATCC 700700 / DSM 12829 / CIP 107037 / JCM 12360 / KCTC 9906 / NCIMB 13794 / A6) OX=452863 GN=ctaB PE=3 SV=1
371	A1KIP0	COXX_MYCBP Protoheme IX farnesyltransferase OS=*Mycobacterium bovis* (strain BCG / Pasteur 1173P2) OX=410289 GN=ctaB PE=3 SV=1
372	A1SJR1	COXX_NOCSJ Protoheme IX farnesyltransferase OS=*Nocardioides* sp. (strain ATCC BAA-499 / JS614) OX=196162 GN=ctaB PE=3 SV=1
373	A1R6I0	COXX_PAEAT Protoheme IX farnesyltransferase OS=*Paenarthrobacter aurescens* (strain TC1) OX=290340 GN=ctaB PE=3 SV=1
374	A0QHX0	COXX_MYCA1 Protoheme IX farnesyltransferase OS=*Mycobacterium avium* (strain 104) OX=243243 GN=ctaB PE=3 SV=2
375	Q0S0I5	COXX_RHOJR Protoheme IX farnesyltransferase OS=*Rhodococcus jostii* (strain RHA1) OX=101510 GN=ctaB PE=3 SV=2
376	Q5YTS1	COXX_NOCFA Protoheme IX farnesyltransferase OS=*Nocardia farcinica* (strain IFM 10152) OX=247156 GN=ctaB PE=3 SV=1
377	Q6AF34	COXX_LEIXX Protoheme IX farnesyltransferase OS=*Leifsonia xyli subsp. xyli* (strain CTCB07) OX=281090 GN=ctaB PE=3 SV=1
378	Q6A7G0	COXX_CUTAK Protoheme IX farnesyltransferase OS=*Cutibacterium acnes* (strain DSM 16379 / KPA171202) OX=267747 GN=ctaB PE=3 SV=2
379	A4QEE9	COXX_CORGB Protoheme IX farnesyltransferase OS=*Corynebacterium glutamicum* (strain R) OX=340322 GN=ctaB PE=3 SV=1
380	A9WT38	COXX_RENSM Protoheme IX farnesyltransferase OS=*Renibacterium salmoninarum* (strain ATCC 33209 / DSM 20767 / JCM 11484 / NBRC 15589 / NCIMB 2235) OX=288705 GN=ctaB PE=3 SV=1
381	C1AN96	COXX_MYCBT Protoheme IX farnesyltransferase OS=*Mycobacterium bovis* (strain BCG / Tokyo 172 / ATCC 35737 / TMC 1019) OX=561275 GN=ctaB PE=3 SV=1
382	A0PPP7	COXX_MYCUA Protoheme IX farnesyltransferase OS=*Mycobacterium ulcerans* (strain Agy99) OX=362242 GN=ctaB PE=3 SV=1
383	A0QWY2	COXX_MYCS2 Protoheme IX farnesyltransferase OS=*Mycolicibacterium smegmatis* (strain ATCC 700084 / mc(2)155) OX=246196 GN=ctaB PE=3 SV=1
384	A1T8M8	COXX_MYCVP Protoheme IX farnesyltransferase OS=*Mycolicibacterium vanbaalenii* (strain DSM 7251 / JCM 13017 / NRRL B-24157 / PYR-1) OX=350058 GN=ctaB PE=3 SV=1
385	A1QRH1	COXX_MYCTF Protoheme IX farnesyltransferase OS=*Mycobacterium tuberculosis* (strain F11) OX=336982 GN=ctaB1 PE=3 SV=1
386	A5U2F4	COXX_MYCTA Protoheme IX farnesyltransferase OS=*Mycobacterium tuberculosis* (strain ATCC 25177 / H37Ra) OX=419947 GN=ctaB PE=3 SV=1
387	Q47ND5	COXX_THEFY Protoheme IX farnesyltransferase OS=*Thermobifida fusca* (strain YX) OX=269800 GN=ctaB PE=3 SV=1
388	A0JWQ8	COXX_ARTS2 Protoheme IX farnesyltransferase OS=*Arthrobacter* sp. (strain FB24) OX=290399 GN=ctaB PE=3 SV=1
389	B1MC66	COXX_MYCA9 Protoheme IX farnesyltransferase OS=*Mycobacteroides abscessus* (strain ATCC 19977 / DSM 44196 / CIP 104536 / JCM 13569 / NCTC 13031 / TMC 1543) OX=561007 GN=ctaB PE=3 SV=1
390	A4F9B0	COXX1_SACEN Protoheme IX farnesyltransferase 1 OS=*Saccharopolyspora erythraea* (strain ATCC 11635 / DSM 40517 / JCM 4748 / NBRC 13426 / NCIMB 8594 / NRRL 2338) OX=405948 GN=ctaB1 PE=3 SV=2
391	A4X9F8	COXX_SALTO Protoheme IX farnesyltransferase OS=*Salinispora tropica* (strain ATCC BAA-916 / DSM 44818 / CNB-440) OX=369723 GN=ctaB PE=3 SV=2
392	Q83GF8	COXX_TROWT Protoheme IX farnesyltransferase OS=*Tropheryma whipplei* (strain Twist) OX=203267 GN=ctaB PE=3 SV=1
393	A8KYS6	COXX_FRASN Protoheme IX farnesyltransferase OS=*Frankia* sp. (strain EAN1pec) OX=298653 GN=ctaB PE=3 SV=1
394	P9WFR7	COXX_MYCTU Protoheme IX farnesyltransferase OS=*Mycobacterium tuberculosis* (strain ATCC 25618 / H37Rv) OX=83332 GN=ctaB PE=3 SV=1
395	P9WFR6	COXX_MYCTO Protoheme IX farnesyltransferase OS=*Mycobacterium tuberculosis* (strain CDC 1551 / Oshkosh) OX=83331 GN=ctaB PE=3 SV=1
396	Q829U3	COXX_STRAW Protoheme IX farnesyltransferase OS=*Streptomyces avermitilis* (strain ATCC 31267 / DSM 46492 / JCM 5070 / NBRC 14893 / NCIMB 12804 / NRRL 8165 / MA-4680) OX=227882 GN=ctaB

		PE=3 SV=2
397	A8LW09	COXX_SALAI Protoheme IX farnesyltransferase OS=*Salinispora arenicola* (strain CNS-205) OX=391037 GN=ctaB PE=3 SV=1
398	Q9XAC2	COXX_STRCO Protoheme IX farnesyltransferase OS=*Streptomyces coelicolor* (strain ATCC BAA-471 / A3(2) / M145) OX=100226 GN=ctaB PE=3 SV=2
399	A4TC38	COXX_MYCGI Protoheme IX farnesyltransferase OS=*Mycolicibacterium gilvum* (strain PYR-GCK) OX=350054 GN=ctaB PE=3 SV=1
400	B0REI2	COXX_CLAMS Protoheme IX farnesyltransferase OS=*Clavibacter michiganensis* subsp. *sepedonicus* (strain ATCC 33113 / DSM 20744 / JCM 9667 / LMG 2889 / C-1) OX=31964 GN=ctaB PE=3 SV=1
401	Q8FT77	COXX_COREF Protoheme IX farnesyltransferase OS=*Corynebacterium efficiens* (strain DSM 44549 / YS-314 / AJ 12310 / JCM 11189 / NBRC 100395) OX=196164 GN=ctaB PE=3 SV=2
402	B1W108	COXX_STRGG Protoheme IX farnesyltransferase OS=*Streptomyces griseus subsp. griseus* (strain JCM 4626 / NBRC 13350) OX=455632 GN=ctaB PE=3 SV=2
403	Q6NH44	COXX_CORDI Protoheme IX farnesyltransferase OS=*Corynebacterium diphtheriae* (strain ATCC 700971 / NCTC 13129 / Biotype gravis) OX=257309 GN=ctaB PE=3 SV=2
404	A0LTZ0	COXX_ACIC1 Protoheme IX farnesyltransferase OS=*Acidothermus cellulolyticus* (strain ATCC 43068 / 11B) OX=351607 GN=ctaB PE=3 SV=1
405	A6WC46	COXX_KINRD Protoheme IX farnesyltransferase OS=*Kineococcus radiotolerans* (strain ATCC BAA-149 / DSM 14245 / SRS30216) OX=266940 GN=ctaB PE=3 SV=1
406	Q8NQ66	COXX_CORGL Protoheme IX farnesyltransferase OS=*Corynebacterium glutamicum* (strain ATCC 13032 / DSM 20300 / BCRC 11384 / JCM 1318 / LMG 3730 / NCIMB 10025) OX=196627 GN=ctaB PE=3 SV=1
407	Q9CCN4	COXX_MYCLE Protoheme IX farnesyltransferase OS=*Mycobacterium leprae* (strain TN) OX=272631 GN=ctaB PE=3 SV=2
408	Q741B3	COXX_MYCPA Protoheme IX farnesyltransferase OS=*Mycolicibacterium paratuberculosis* (strain ATCC BAA-968 / K-10) OX=262316 GN=ctaB PE=3 SV=1
409	Q0RH20	COXX_FRAAA Protoheme IX farnesyltransferase OS=*Frankia alni* (strain ACN14a) OX=326424 GN=ctaB PE=3 SV=2
410	Q2JCG7	COXX_FRACC Protoheme IX farnesyltransferase OS=*Frankia casuarinae* (strain DSM 45818 / CECT 9043 / CcI3) OX=106370 GN=ctaB PE=3 SV=1
411	Q9Y5Z9	UBIA1_HUMAN UbiA prenyltransferase domain-containing protein 1 OS=*Homo sapiens* OX=9606 GN=UBIAD1 PE=1 SV=1
412	Q96H96	COQ2_HUMAN 4-hydroxybenzoate polyprenyltransferase, mitochondrial OS=*Homo sapiens* OX=9606 GN=COQ2 PE=1 SV=1
413	E7FB98	UBIA1_DANRE UbiA prenyltransferase domain-containing protein 1 OS=*Danio rerio* OX=7955 GN=ubiad1 PE=1 SV=1
414	P0AGK1	UBIA_ECOLI 4-hydroxybenzoate octaprenyltransferase OS=*Escherichia coli* (strain K12) OX=83333 GN=ubiA PE=1 SV=1
415	Q96H96	COQ2_HUMAN 4-hydroxybenzoate polyprenyltransferase, mitochondrial OS=*Homo sapiens* OX=9606 GN=COQ2 PE=1 SV=1
416	P32378	COQ2_YEAST 4-hydroxybenzoate polyprenyltransferase, mitochondrial OS=*Saccharomyces cerevisiae* (strain ATCC 204508 / S288c) OX=559292 GN=COQ2 PE=1 SV=1
417	Q9VHS7	COQ2_DROME 4-hydroxybenzoate polyprenyltransferase, mitochondrial OS=*Drosophila melanogaster* OX=7227 GN=Coq2 PE=2 SV=1
418	Q93YP7	COQ2_ARATH 4-hydroxybenzoate polyprenyltransferase, mitochondrial OS=*Arabidopsis thaliana* OX=3702 GN=PPT1 PE=2 SV=1
419	Q10252	COQ2_SCHPO 4-hydroxybenzoate polyprenyltransferase, mitochondrial OS=*Schizosaccharomyces pombe* (strain 972 / ATCC 24843) OX=284812 GN=ppt1 PE=1 SV=2
420	Q298G6	COQ2_DROPS 4-hydroxybenzoate polyprenyltransferase, mitochondrial OS=*Drosophila pseudoobscura* pseudoobscura OX=46245 GN=Coq2 PE=3 SV=1
421	Q54U71	COQ2_DICDI 4-hydroxybenzoate polyprenyltransferase, mitochondrial OS=*Dictyostelium discoideum* OX=44689 GN=coq2 PE=3 SV=1
422	Q66JT7	COQ2_MOUSE 4-hydroxybenzoate polyprenyltransferase, mitochondrial OS=*Mus musculus* OX=10090 GN=Coq2 PE=2 SV=2
423	Q8I7J4	COQ2_CAEEL 4-hydroxybenzoate polyprenyltransferase, mitochondrial OS=*Caenorhabditis elegans* OX=6239 GN=coq-2 PE=3 SV=2
424	Q16QL3	COQ2_AEDAE 4-hydroxybenzoate polyprenyltransferase, mitochondrial OS=*Aedes aegypti* OX=7159 GN=coq2 PE=3 SV=1
425	Q499N4	COQ2_RAT 4-hydroxybenzoate polyprenyltransferase, mitochondrial OS=*Rattus norvegicus* OX=10116 GN=Coq2 PE=2 SV=1
426	Q2KIQ4	COQ2_BOVIN 4-hydroxybenzoate polyprenyltransferase, mitochondrial OS=*Bos taurus* OX=9913 GN=COQ2 PE=2 SV=1
427	A1JHN0	HSTC_CHLRE Homogentisate solanesyltransferase, chloroplastic OS=*Chlamydomonas reinhardtii* OX=3055 GN=HST PE=1 SV=1
428	Q8VWJ1	HPT1_ARATH Homogentisate phytyltransferase 1, chloroplastic OS=*Arabidopsis thaliana* OX=3702 GN=HPT1 PE=1 SV=1
429	Q1ACB3	HSTC_ARATH Homogentisate solanesyltransferase, chloroplastic OS=*Arabidopsis thaliana* OX=3702 GN=HST PE=1 SV=1
430	A0A077K8G3	CGT1A_CITLI Coumarin 8-geranyltransferase 1, chloroplastic OS=*Citrus limon* OX=2708 GN=ClPT1 PE=1 SV=1
431	Q7XB14	HGGT_HORVU Homogentisate geranylgeranyltransferase OS=*Hordeum vulgare* OX=4513 GN=HGGT PE=1 SV=1
432	P73726	HGGT_SYNY3 Homogentisate phytyltransferase OS=*Synechocystis* sp. (strain PCC 6803 / Kazusa) OX=1111708 GN=slr1736 PE=1 SV=1
433	A0A077K9K6	CGT1B_CITLI Coumarin 8-geranyltransferase 1b, chloroplastic OS=*Citrus limon* OX=2708 GN=Cl-PT1b PE=1 SV=1
434	Q0DAK7	HGGT_ORYSJ Homogentisate geranylgeranyltransferase, chloroplastic OS=*Oryza sativa* subsp. *japonica* OX=39947 GN=HGGT PE=2 SV=2
435	B7FA90	HPT1_ORYSJ Probable homogentisate phytyltransferase 1, chloroplastic OS=*Oryza sativa* subsp. *japonica* OX=39947 GN=HPT1 PE=2 SV=1

436	Q0D576	HPT2_ORYSJ Probable homogentisate phytyltransferase 2, chloroplastic OS=*Oryza sativa* subsp. *japonica* OX=39947 GN=HPT2 PE=3 SV=2
437	Q7XB13	HGGT_WHEAT Homogentisate geranylgeranyltransferase OS=*Triticum aestivum* OX=4565 GN=HGGT PE=2 SV=1
438	P9WFR5	DPPRS_MYCTU Decaprenyl-phosphate phosphoribosyltransferase OS=*Mycobacterium tuberculosis* (strain ATCC 25618 / H37Rv) OX=83332 GN=Rv3806c PE=1 SV=1
439	P9WFR4	DPPRS_MYCTO Decaprenyl-phosphate phosphoribosyltransferase OS=*Mycobacterium tuberculosis* (strain CDC 1551 / Oshkosh) OX=83331 GN=MT3913 PE=3 SV=1
440	A0R626	DPPRS_MYCS2 Decaprenyl-phosphate phosphoribosyltransferase OS=*Mycolicibacterium smegmatis* (strain ATCC 700084 / mc(2)155) OX=246196 GN=MSMEG_6401 PE=3 SV=1
441	Q8NLQ9	DPPRS_CORGL Decaprenyl-phosphate phosphoribosyltransferase OS=*Corynebacterium glutamicum* (strain ATCC 13032 / DSM 20300 / BCRC 11384 / JCM 1318 / LMG 3730 / NCIMB 10025) OX=196627 GN=cg3189 PE=3 SV=1
442	Q5W6H5	CHLG_ORYSJ Chlorophyll synthase, chloroplastic OS=*Oryza sativa subsp. japonica* OX=39947 GN=CHLG PE=2 SV=1
443	Q9M3W5	CHLG_AVESA Chlorophyll synthase, chloroplastic OS=*Avena sativa* OX=4498 GN=CHLG PE=1 SV=1
444	Q38833	CHLG_ARATH Chlorophyll synthase, chloroplastic OS=*Arabidopsis thaliana* OX=3702 GN=CHLG PE=2 SV=1
445	A4F5C0	A4F5C0_STIAU Prenyltransferase OS=*Stigmatella aurantiaca* OX=41 GN=auaA PE=4 SV=1

* Accession numbers/ IDs are taken from PATRIC database (1-114) and UniProt database (115-445).

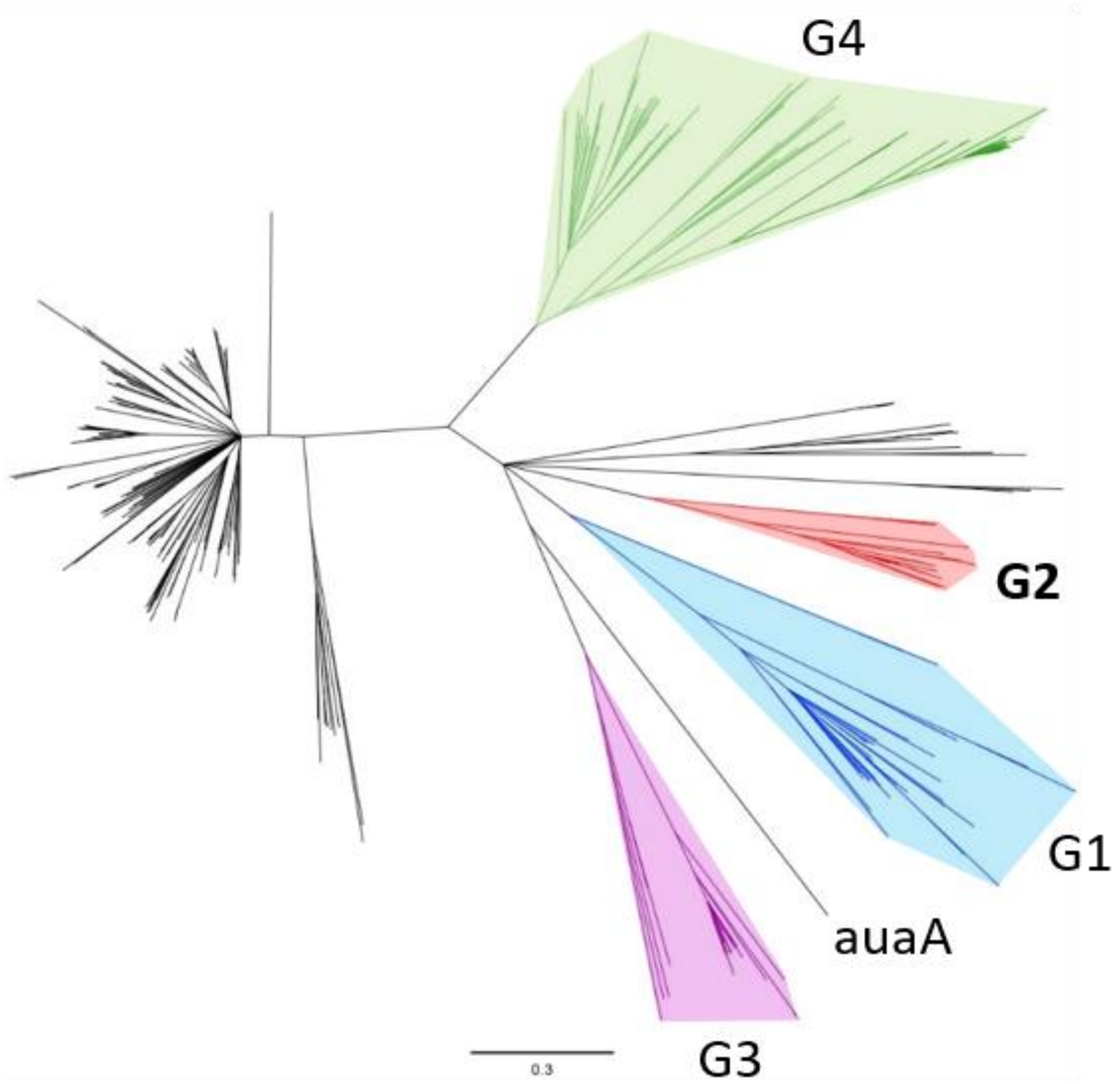

Figure S1: Phylogenetic tree of Ptases from marine Flavobacteria, *Sacharomonospora* and Uniprot database. G1: 1,4-dihydroxy-2-naphthoate polyprenyltransferase (blue), G2: UbiA-like PTases (red), G3: (S)-2,3-di-O-geranylgeranylglyceryl phosphate synthase (purple), G4: Protoheme IX farnesyltransferase (green). Phylogenetic tree was constructed using clustal omega multiple alignment and neighbor-joining method. 500 replicates were used for bootstrap resampling method.

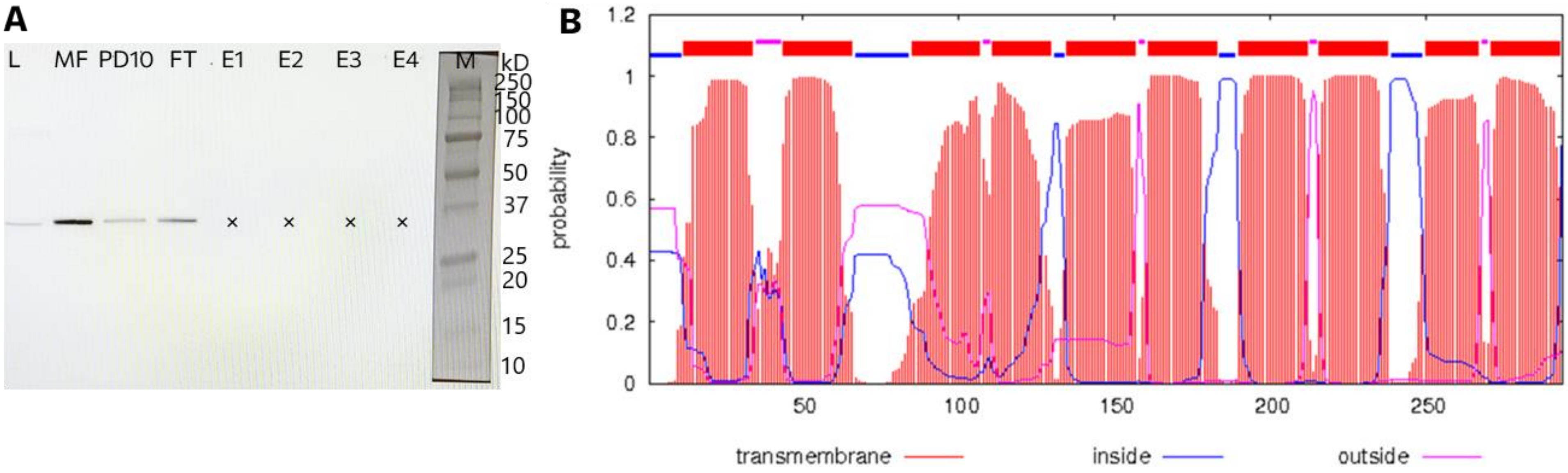

Figure S2: A) Heterologous expression of G2 UbiA-297 of *Maribacter* sp. MS6 in *E. coli*. Lanes from left to right: Crude lysate (L), membrane fraction (MF), PD10 column flow through (PD10), NiNTA flow through (FT), NiNTA elution1- 100 mM imidazol (E1), NiNTA elution 2-150 mM imidazole (E2), NiNTA elution3- 200 mM imidazole (E3), NiNTA elution4- 300 mM imidazole (E4), Biorad all blue protein marker (M) and size indication of marker in kD. B) TMHMM posterior probabilities for G2 UbiA-297 of *Maribacter* sp. MS6.

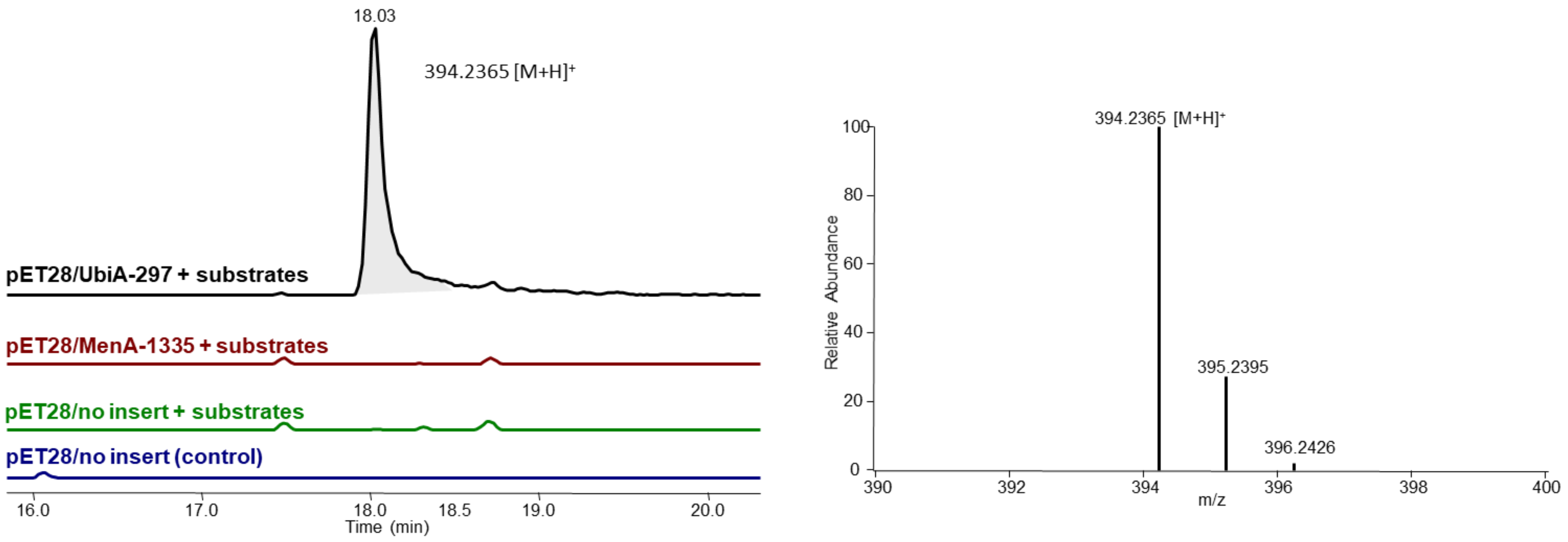

Figure S3: Extracted ion chromatogram (EIC for *m/z* 378.2428) for the in vivo assay extracts of UbiA-297 using 8-HQA and FPP as substrates and HRMS of product peak at RT 18.03 min.

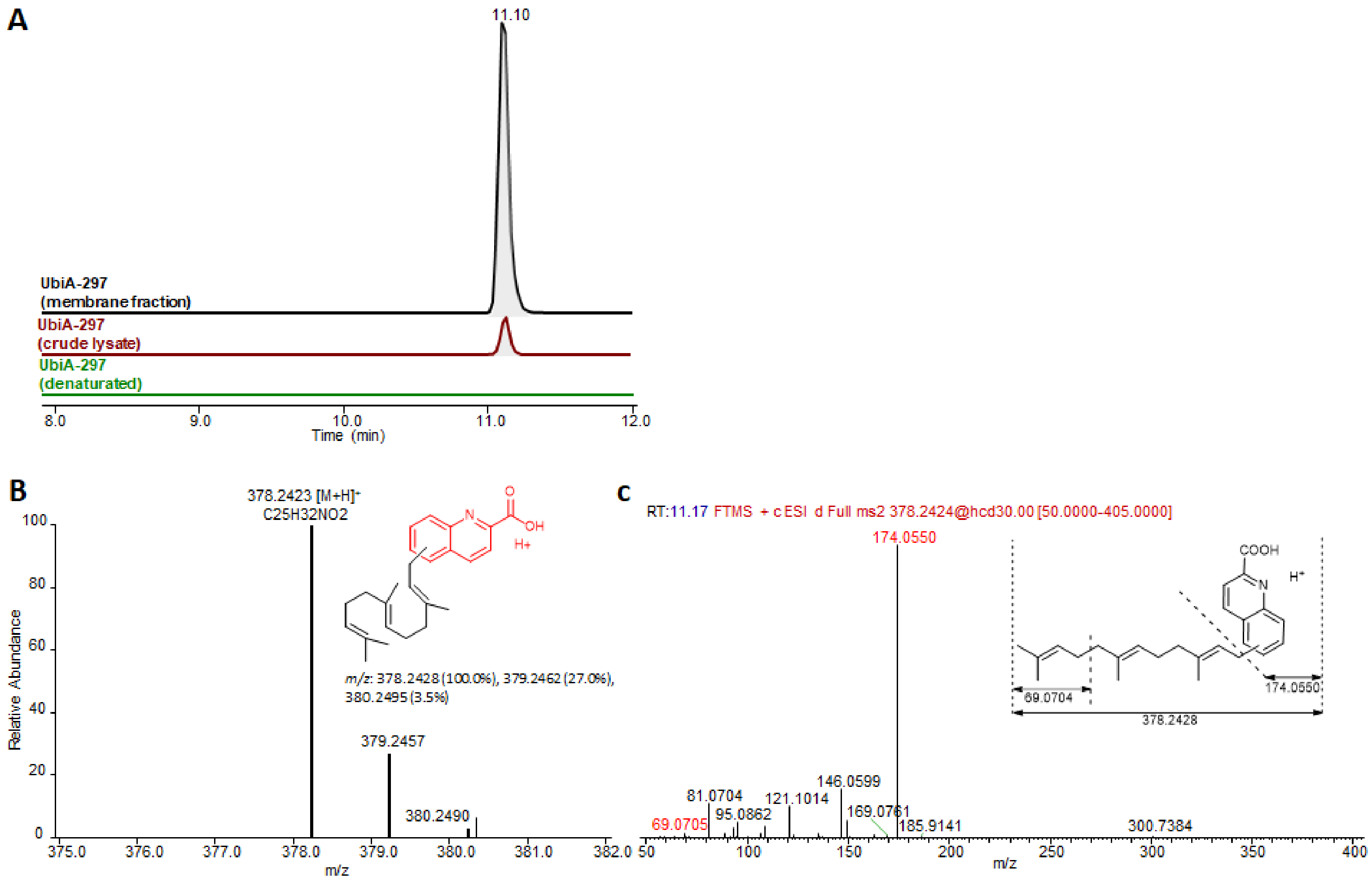

Figure S4: HRMS/MS analysis of the *in vitro* assays using quinaldic acid and FPP as substrates (short gradient, 15 min run). A) Extracted ion chromatogram (EIC for *m/z* 378.2428) for the assay extracts of UbiA-297 membrane fraction, crude protein lysate, and denaturated membrane fraction. Fixed ion intensity scale (1.05E7) was applied to all chromatograms; B) MS-spectrum of the peak at 11.10 min from the membrane fraction assay of UbiA-297; C) MS/MS fragmentation of prenylated product at *m/z* 378.2424. (Putative fragmentation pattern of farnesylated quinaldic acid is depicted).

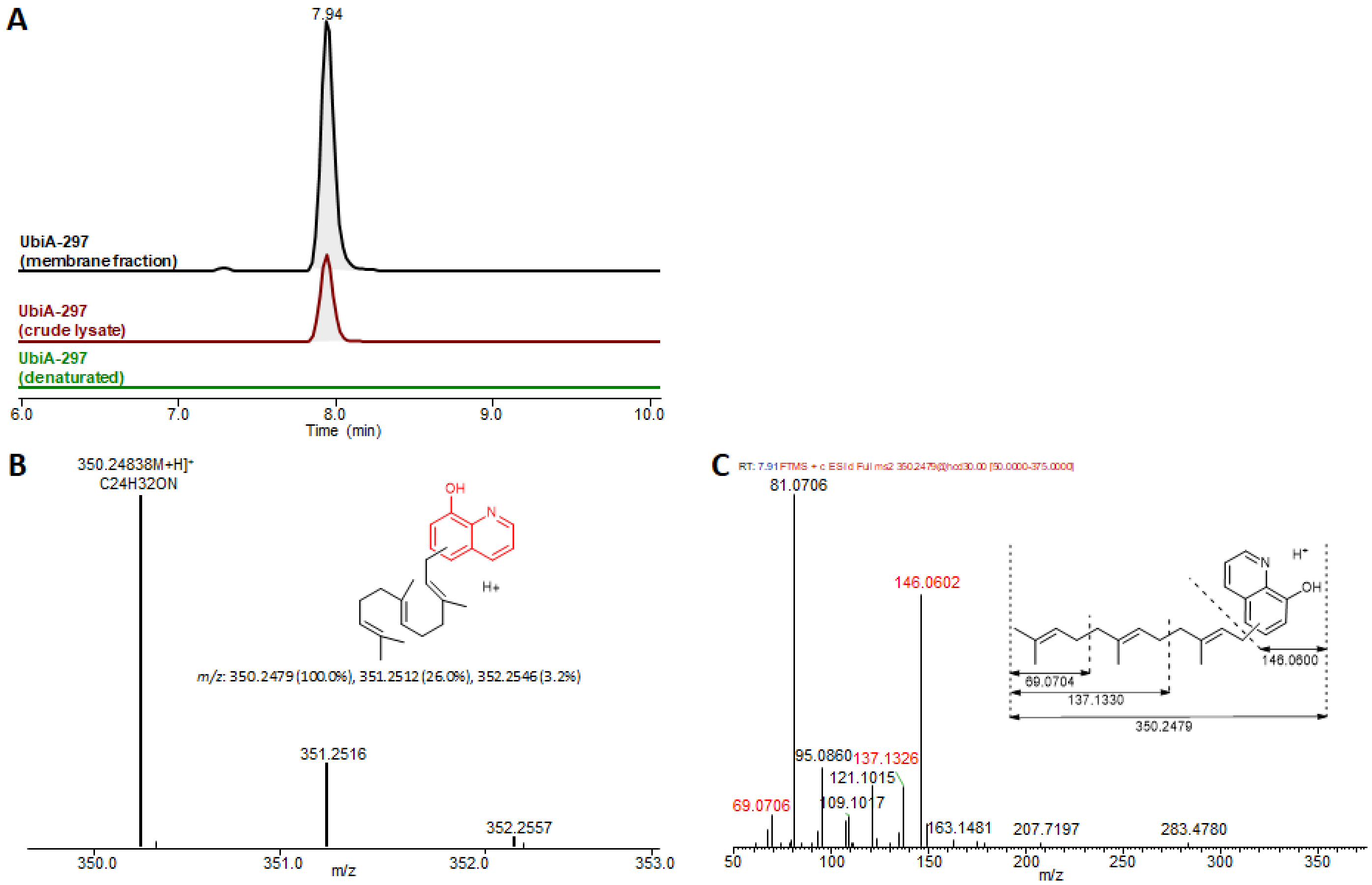

Figure S5: HRMS/MS analysis of the *in vitro* assays using 8-hydroxyquinoline and FPP as substrates (short gradient, 15 min run). A) Extracted ion chromatogram (EIC for *m/z* 350.2479) for the assay extracts of UbiA-297 membrane fraction, crude protein lysate, and denaturated membrane fraction. Fixed ion intensity scale (2.82E6) was applied to all chromatograms; B) MS-spectrum of the peak at 11.10 min from the membrane fraction assay of UbiA-297; C) MS/MS fragmentation of prenylated product at *m/z* 350.2479. (Putative fragmentation pattern of farnesylated 8-hydroxyquinoline is depicted).

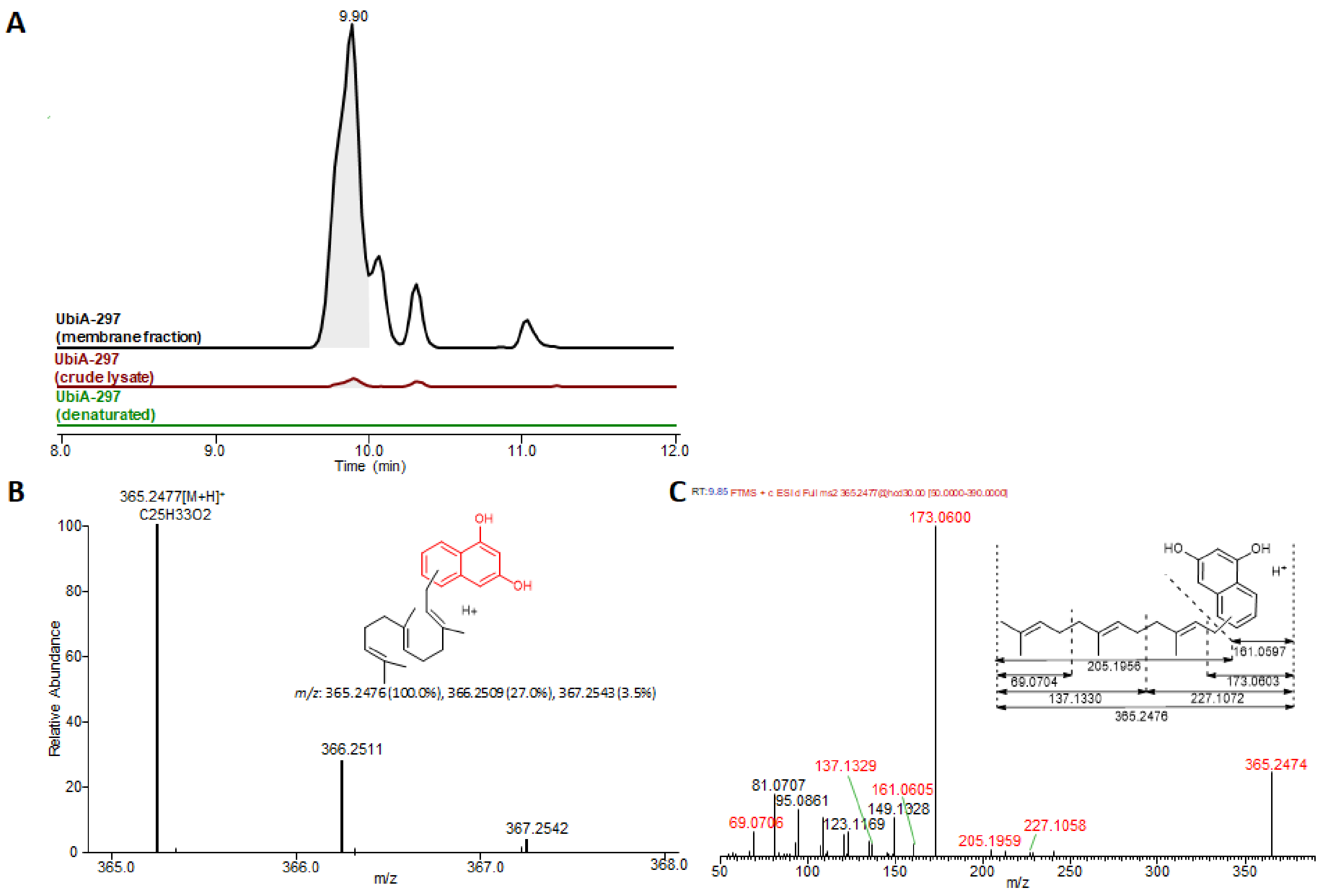

Figure S6: HRMS/MS analysis of the in vitro assays using 1,3-dihydroxynaphtalene and FPP as substrates (short gradient,15 min run). A) Extracted ion chromatogram (EIC for *m/z* 365.2476) for the assay extracts of UbiA-297 membrane fraction, crude protein lysate, and denaturated membrane fraction. Fixed ion intensity scale (2.05E6) was applied to all chromatograms; B) MS-spectrum of the peak at 11.10 min from the membrane fraction assay of UbiA-297; C) MS/MS fragmentation of prenylated product at *m/z* 365.2474. (Putative fragmentation pattern of farnesylated 1,3-dihydroxynaphtalene is depicted).

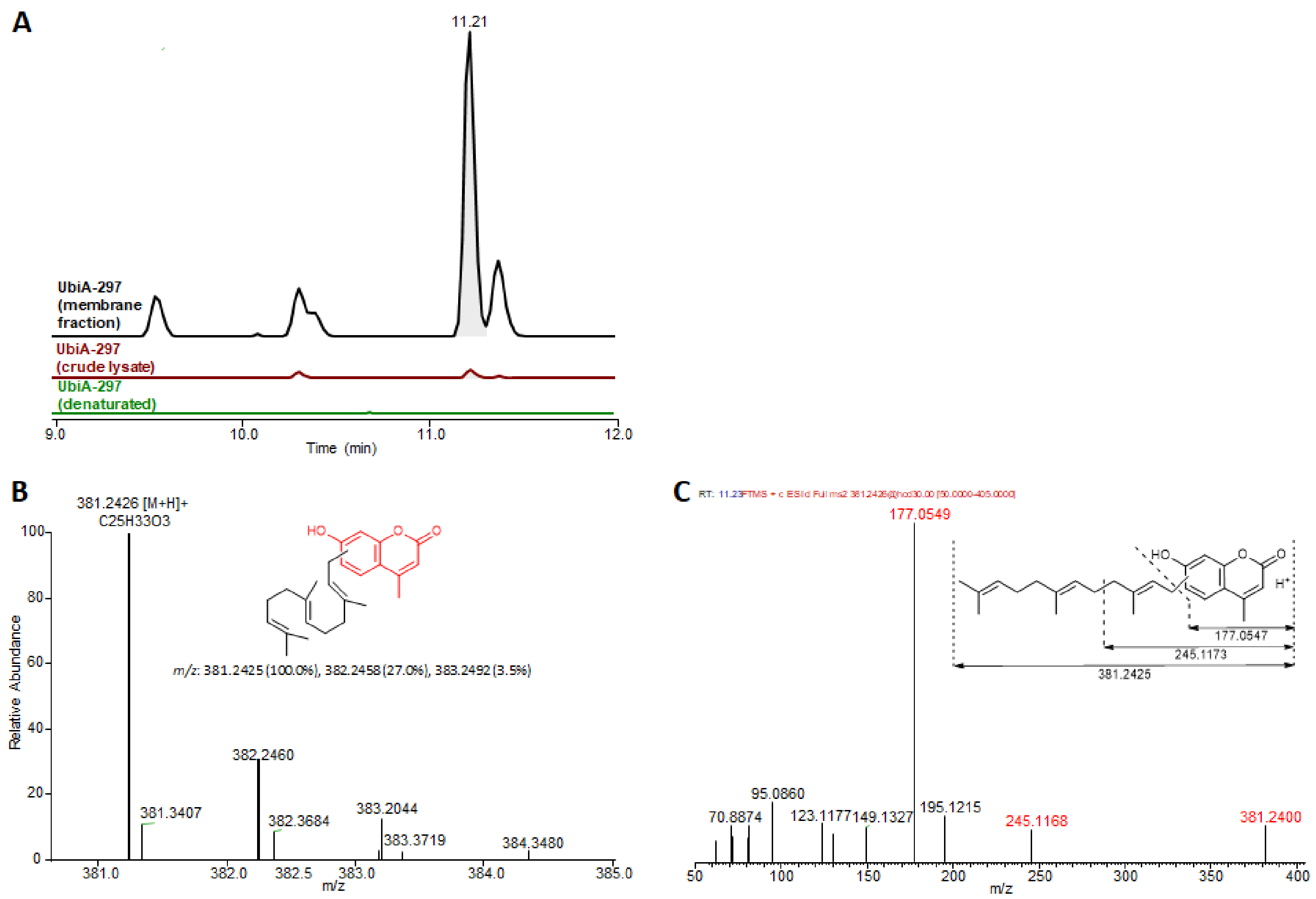

Figure S7: HRMS/MS analysis of the *in vitro* assays using 4-methylumbelliferone and FPP as substrates (short gradient,15 min run). A) Extracted ion chromatogram (EIC for *m/z* 381.2425) for the assay extracts of UbiA-297 membrane fraction, crude protein lysate, and denaturated membrane fraction. Fixed ion intensity scale (7.45E5) was applied to all chromatograms; B) MS-spectrum of the peak at 11.10 min from the membrane fraction assay of UbiA-297; C) MS/MS fragmentation of prenylated product at *m/z* 381.2425. (Putative fragmentation pattern of farnesylated 4-methylumbelliferone is depicted).

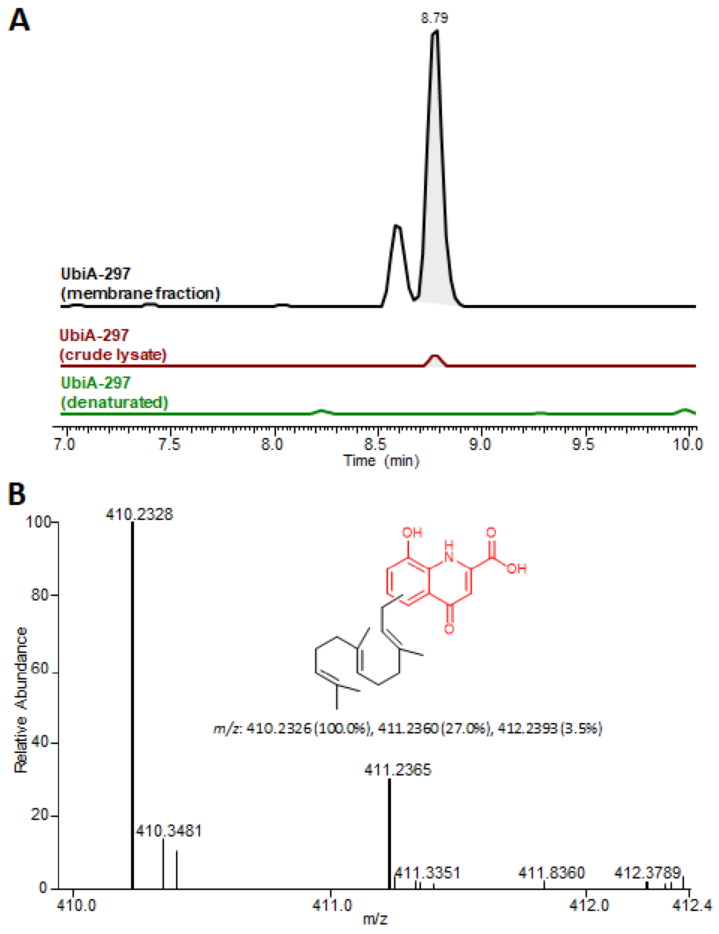

Figure S8: HRMS/MS analysis of the *in vitro* assays using xanthurenic acid and FPP as substrates (short gradient, 15 min run). A) Extracted ion chromatogram (EIC for *m/z* 410.2326) for the assay extracts of UbiA-297 membrane fraction, crude protein lysate, and denaturated membrane fraction. Fixed ion intensity scale (3.14E5) was applied to all chromatograms; B) MS-spectrum of the peak at 11.10 min from the membrane fraction assay of UbiA-297.

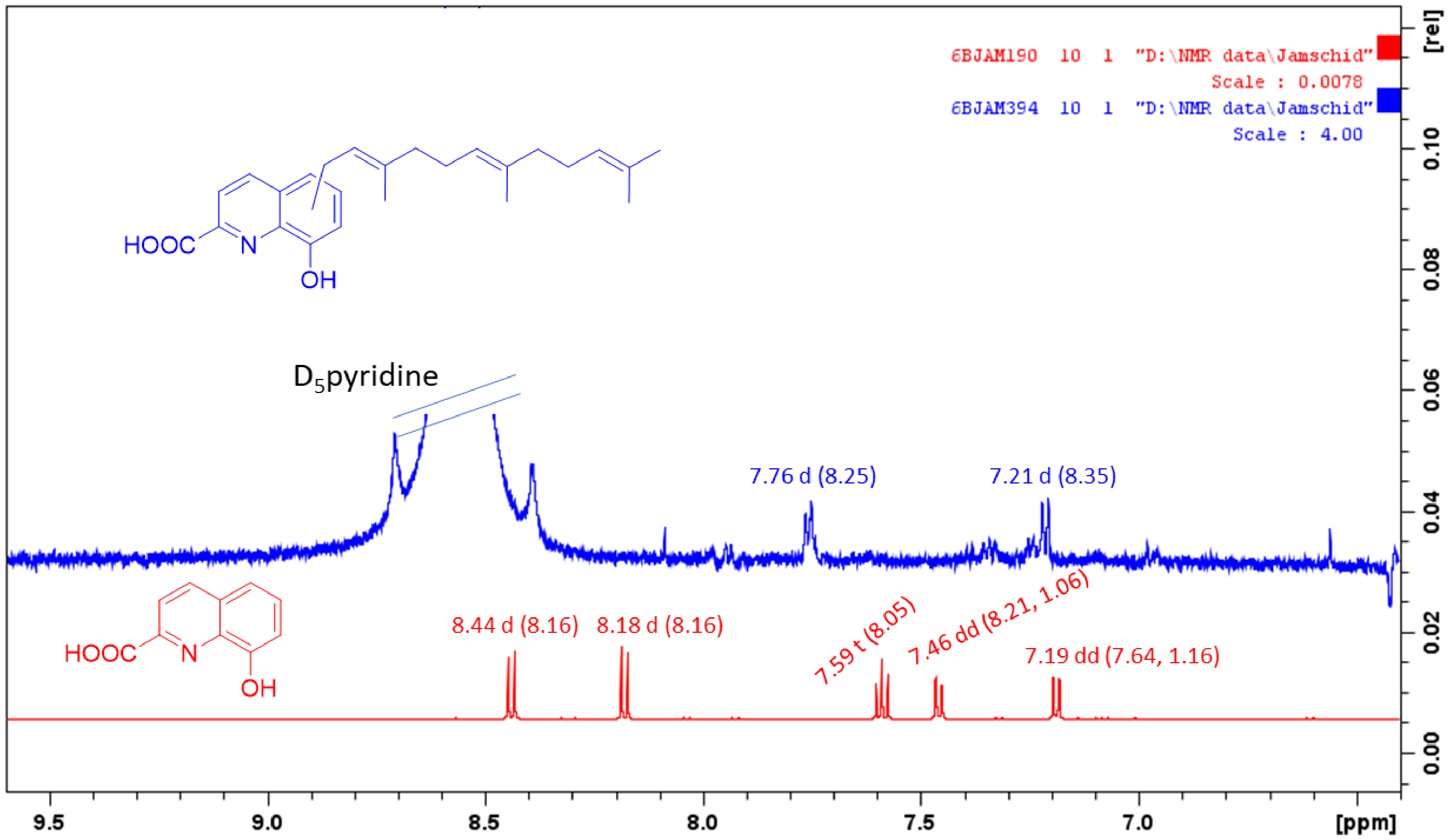

Figure S9: Overlayed ^{1}H NMR spectra of A) isolated product with *m/z* 394.2376 (blue), and B) starting material 8-HQA (red), 600 MHz (*d*5-pyridine).
